# Interference of Polydatin/Resveratrol in the ACE2:Spike Recognition during COVID-19 Infection. A Focus on Their Potential Mechanism of Action through Computational and Biochemical Assays

**DOI:** 10.3390/biom11071048

**Published:** 2021-07-16

**Authors:** Fulvio Perrella, Federico Coppola, Alessio Petrone, Chiara Platella, Daniela Montesarchio, Annarita Stringaro, Giampietro Ravagnan, Maria Pia Fuggetta, Nadia Rega, Domenica Musumeci

**Affiliations:** 1 Department of Chemical Sciences, University of Naples Federico II, 80126 Naples, Italy; fulvio.perrella@unina.it (F.P.); federico.coppola@unina.it (F.C.); alessio.petrone@unina.it (A.P.); chiara.platella@unina.it (C.P.); montesar@unina.it (D.M.); 2 National Center for Drug Research and Evaluation, Italian National Institute of Health, 00161 Rome, Italy; annarita.stringaro@iss.it; 3 Institute of Translational Pharmacology, Consiglio Nazionale delle Ricerche, 00133 Rome, Italy; gprav@unive.it; 4 Centro di Ricerca Interdipartimentale sui Biomateriali, University of Naples Federico II, Piazzale Tecchio, 80125 Naples, Italy; 5 Institute of Biostructures and Bioimages, Consiglio Nazionale delle Ricerche, 80134 Naples, Italy

**Keywords:** SARS-CoV-2, polydatin, resveratrol, molecular docking, protein-binding, ACE2:Spike binding-inhibition

## Abstract

In the search for new therapeutic strategies to contrast SARS-CoV-2, we here studied the interaction of polydatin (PD) and resveratrol (RESV)—two natural stilbene polyphenols with manifold, well known biological activities—with Spike, the viral protein essential for virus entry into host cells, and ACE2, the angiotensin-converting enzyme present on the surface of multiple cell types (including respiratory epithelial cells) which is the main host receptor for Spike binding. Molecular Docking simulations evidenced that both compounds can bind Spike, ACE2 and the ACE2:Spike complex with good affinity, although the interaction of PD appears stronger than that of RESV on all the investigated targets. Preliminary biochemical assays revealed a significant inhibitory activity of the ACE2:Spike recognition with a dose-response effect only in the case of PD.

## 1. Introduction

Coronaviruses (CoV) are a large family of viruses that may cause disease in animals or humans [1,2,3]. They can provoke respiratory infections ranging from the common cold to more severe illnesses [3]. The novel coronavirus, called SARS-CoV-2, which emerged in December 2019 causing coronavirus disease 2019 (COVID-19), can lead to serious, even fatal, disease [4,5,6], and was declared a global pandemic by the World Health Organization on 11 March 2020.

All coronaviruses possess an enveloped, positive-sense, single-stranded RNA genome encoding for 4 structural membrane proteins, i.e., Spike (S), envelope (E), membrane (M) and nucleocapsid (N) proteins [7]. The Spike proteins S are essential for viral entry into host cells, which occurs essentially through binding to the angiotensin-converting enzyme ACE2 [8,9,10,11]. ACE2 is present on the surface of multiple cell types, including respiratory and intestinal epithelial cells, endothelial cells, kidney cells (renal tubules), cerebral neurons, and immune cells, such as alveolar monocytes/macrophages [12,13].

Therefore, bioactive compounds able to inhibit the interaction between the COVID-19 S protein and the ACE2 receptor may be precious drugs for effective antiviral therapeutic strategies [14]. Indeed, human neutralizing antibodies targeting S protein and blocking SARS-CoV-2 cellular entry are promising therapeutic tools [15,16,17,18,19].

After attachment of the virus, a proteolytic enzyme of the host cell, mainly type II transmembrane serine protease TMPRSS2, cleaves and activates the receptor-attached Spike macromolecule [20]. This protease, anchored in the cell membrane near ACE2 receptors, and expressed in the epithelial cell lining of the nose, trachea and distal airways, cleaves SARS-CoV-2 S protein into two subunits, S1 and S2, respectively. The N-terminus of S1 subunit represents the receptor-binding domain (RBD) which binds to ACE2, whereas S2 subunit serves to promote fusion activity via its *C*-terminus [20].

Structural data of the viral proteins, as well as of the main host proteins favouring virus entry in the host cells, are extremely important for the development of compounds able to specifically recognize key regions of these macromolecules, potentially acting as anti-COVID-19 effective drugs. Drugs able to bind key regions of the selected targets with high affinity and specificity could in principle sterically block the binding sites of the viral/host proteins or induce conformational switches in the biomolecules avoiding their correct recognition. Various works have already investigated, experimentally or in silico, the effects of natural compounds or synthetic drugs on COVID-19-related targets [21,22,23,24,25]. Several natural products endowed with significant biological activities, especially extracted from plants, have been thus identified as potentially able to contrast the dissemination of Coronavirus and, at the same time, enhance immunity, stimulating further screenings to discover new candidate drugs.

**Figure 1:**
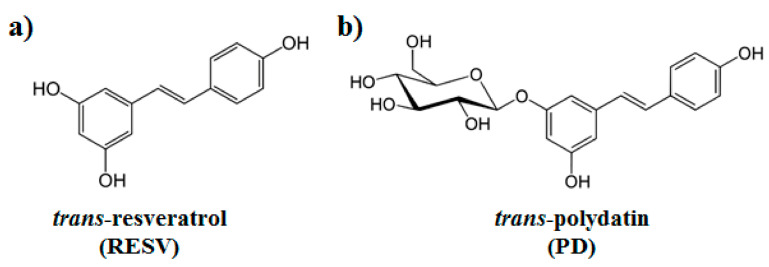
Chemical structures of (**a**) trans-resveratrol (RESV) and (**b**) trans-polydatin (PD).

Natural polyphenols are an abundant and widely distributed family of bioactive molecules, whose structure is generally constituted by one or more aromatic rings carrying one or more hydroxyl groups [26]. Two natural stilbene polyphenols that have attracted much attention, especially for their manifold biological properties, are trans-resveratrol (here named RESV, 3,5,4′-trihydroxystilbene) [27] and trans-polydatin (here named PD, 3,5,4′-trihydroxystilbene-3-β-D-glucoside, Figure 1) [28]. These polyphenols were originally isolated from the root and rhizome of Polygonum cuspidatum, a plant used in traditional Chinese medicine for its analgesic, antipyretic and diuretic properties. Resveratrol is a phytoalexin produced by more than 70 plants in response to various stresses and is found in a variety of foods, including red grapes, peanuts, pistachios, red wine, blueberries, cranberries, and even cocoa and dark chocolate [29]. Polydatin is a glycosylated form of RESV and the most abundant derivative of resveratrol in nature [30].

Many studies have been carried out on the beneficial effects of these polyphenols on the human body (e.g., anti-oxidant, anti-inflammatory, antitumor, antiviral, neuroprotective, hepatoprotective and ischemia preventing activities), and on their mechanisms of action [27,28,31,32,33].

Analogously to other polyphenols, RESV has limited bioavailability and poor water solubility [34]. On the other hand, PD displays higher water solubility and metabolic stability, as well as better oral absorption than RESV and is used in clinics with no side effects [35,36].

These compounds were recently proposed as potential drugs against COVID-19-related targets as indicated by preliminary in silico studies and cellular assays [37,38,39]. Furthermore, polydatin and resveratrol treatments could be beneficial for COVID-19 infection also due to their anti-inflammatory activities particularly in the respiratory tract [40,41,42,43,44,45,46,47,48,49].

On these bases, we here investigated—by means of detailed in silico studies and preliminary biochemical assays—the potential of RESV and PD to bind ACE2 and/or Spike proteins interfering with their interaction, essential for virus host-cell entry. To the best of our knowledge, this is the first report exploring, with preliminary experimental assays, the interference of PD/RESV on the binding of a COVID-19 key protein to a host target.

In particular, we here studied the interactions of PD and RESV with both Spike and ACE2 as separated proteins as well as with their complex through a molecular docking-based computational approach, using the available molecular structures as deposited in the PDB database. Furthermore, preliminary biochemical assays, i.e., ELISA-like assays employing the target recombinant proteins (Spike S1 subunit and ACE2) and the tested small-molecules, were performed to evaluate the ability of PD/RESV to inhibit/block the ACE2 recognition by Spike. It is worth underlining that ACE2, even if identified as the entrance receptor for SARS-CoV2 in the host cell, is implicated also in a wide range of physiological processes. Ideally, a drug should interact with the key host receptor blocking only the pathological pathway (i.e., the interaction with Spike). In our case, since it is well known that RESV and PD are non-toxic even in high dosage—and indeed they are used as nutraceuticals and drugs in various applications—we are confident that they will not cause side effects connected with their interactions with ACE2.

## 2. Materials and Methods

### 2.1. Computational Details

Cryogenic electron microscopy (cryo-EM) structures of SARS-CoV-2 Spike protein, human ACE2 receptor and Spike protein receptor-binding domain complexed with ACE2 were retrieved from the ProteinDataBank archive [PDB IDs: 6VSB [50], 6M18 [51], 6VW1 [10]. Only one heterodimeric pair of ACE2 and Spike (chains A and E, respectively) was retained in the ACE2:Spike complex. Polar hydrogens were added by using AutoDockTools [52]. Glycosylation sites [consisting of β-D-mannopyranose (BMA) and β-D-(acetylamino)-2-deoxy-glucopyranose (NAG) residues] were included. All remaining co-crystallized ligands not covalently bound, such as crystallographic water molecules, zinc and chloride ions and 1,2-ethanediol were removed from the structure.

RESV and PD chemical structures were built with the GaussView 6 [53] molecular editor and fully optimized in gas phase without any constraint at Density Functional Theory (DFT) level employing the B3LYP functional (Becke three-parameter hybrid functional combined with Lee-Yang-Parr correlation functional [54,55]), with a 6–311 + G (d,p) split-valence triple-zeta basis set adding diffuse functions on heavy atoms and polarization functions on all atoms. Harmonic vibrational frequencies analysis was also carried out to ensure the true local minimum energy nature of both structures. These calculations were performed by using the Gaussian16 electronic structure software package [56]. The optimized RESV and PD three-dimensional structures are reported in Figure S1.

Molecular docking simulations were carried out with AutoDock Vina [57]. The initial set-up, comprising the generation of the pdbqt files of ligands and proteins, the configuration file and the choice of the grid box (centre and dimensions), was carefully performed by using the graphical interface of AutoDockTools.

All hosts investigated in this work were treated as rigid species. All torsional degrees of freedom were kept free for both guests, i.e., PD and RESV, except rotations around the ethylene bridge linking the two phenyl rings in these molecules. The potential binding sites were detected through a virtual screening by using Fpocket server suite [58,59] which analyses proteins surface and identifies cavities and pockets which could host a putative ligand. FPocket is a cavity prediction algorithm widely used for the identification of binding sites and molecular docking simulations. Compared to other similar algorithms, it is very fast for large proteins, stands out in accuracy and does not require any prior protein preparation or parameters optimization [60,61,62,63,64,65,66,67,68].

No known experimental structure describing a complex between S-protein Receptor Binding Domain (located in the most external region of Spike structure and so highly fluctuating) and a small ligand exists to date. Consequently, no well-defined binding sites are known for Spike RBD. A classical docking validation through re-docking an experimental ligand-protein complex cannot be therefore carried out in this case. In contrast to a “focused” docking targeted to an already known binding site, our docking calculations can be considered as “blind” docking [69,70,71,72,73,74].

Cubic grids of up to 30 Å per side were placed to cover all the potential ligand-binding sites on the Spike/ACE2 interaction surface. 20 poses were saved for each run. A high exhaustiveness parameter (which controls the search accuracy inside the chosen box) was selected. For each investigated protein surface region, clustering of docked poses was performed according to their RMSD (5 Å bins) and visual inspection. Nevertheless, only the top-ranked pose has been retained and discussed in detail, assuming a −6.50 kcal/mol docking score threshold. Intermolecular interactions with the surrounding amino acid residues such as hydrogen bonds and hydrophobic contacts were evaluated for these poses and plotted using LigPlot+ [75].

### 2.2. Biochemical Assays

#### 2.2.1. Instrumentations

The reactions on the plate were incubated on an orbital undulating shaker (Sunflower 3D Mini-Shaker, Biosan, Riga, Latvia; purchased from Stereoglass s.r.l, Perugia, Italy). Luminescence was measured using a 96-well plate reader (GloMax-96 microplate luminometer; Promega Italia s.r.l, Milan, Italy).

#### 2.2.2. Chemicals

Polydatin and resveratrol were kindly provided by Glures Srl, Spin Off of CNR-Italy.

ACE2:SARS-CoV-2 Spike S1 inhibitor screening assay kit (BPS bioscience, San Diego, CA, USA) and MAXTAG 10× PBS (phosphate-buffered saline; Rockland Inc., Limerick, PA, USA) were purchased from tebu-bio s.r.l. (Milan, Italy). The kit contained the following reactants: SARS-CoV-2 Spike S1, Fc Fusion, Avi-tag, Biotin-labelled (0.65 mg/mL); ACE2 His-Tag (1 mg/mL; here named ACE2-His); Streptavidin-HRP (horseradish peroxidase); 3× Immuno Buffer 1 (IB1); Blocking Buffer 2 (BB2); ELISA ECL (enhanced chemiluminescence) substrate A (ECL-A)/ELISA ECL substrate B (ECL-A); Nickel-coated 96-well white microplate. Dimethyl sulfoxide (DMSO) and nuclease-free sterile water were purchased from VWR.

#### 2.2.3. Proteins and Inhibitors Solutions Preparation

A single-use aliquot of concentrated ACE2-His was diluted to 1 μg with 1× PBS. A single-use aliquot of concentrated SARS-CoV-2 Spike S1-Biotin was diluted to 5 ng/μL (approximately 50 nM) with 1× IB1. Streptavidin-HRP was diluted 1000-fold with BB2. Weighed amounts of RESV and PD were dissolved in DMSO to obtain concentrated solutions of the polyphenols (20 mM); this solution was then diluted with 1x PBS to the proper concentration in order not to exceed 1% DMSO in the final incubation solution on the well.

#### 2.2.4. Spike-ACE2 Binding Assay

First, ACE2 was immobilized on the Nickel-coated plate by adding 50 μL of ACE2-His diluted solution to each well of the plate and incubating at room temperature for 2 h under slow shaking. Then, the unreacted Ni-sites were blocked with BB2, as reported in the assay-kit datasheet.

Next, three different treatments were tested to optimize the protocol: (1) Treatment A, pre-incubation of RESV/PD with ACE2-coated plate; (2) Treatment B, pre-incubation of RESV/PD with Spike S1 in solution; (3) Treatment C, pre-incubation of Spike S1 with ACE2-coated plate. In Treatment A, 20 μL of 1× IB1 were added to each well, followed by 10 μL of inhibitor solutions for the samples wells, and 10 μL of the same solution without inhibitor (inhibitor buffer) for the “Positive Control” and “Blank”. Incubation at room temperature for 30 min under slow shaking was performed. Then, 20 μL of SARS-CoV-2 Spike S1-Biotin solution were added to the samples and positive wells, whereas 20 μL of only 1× IB1 were added to the “Blank” wells, incubating at room temperature for 1 h under slow shaking. In Treatment B, 20 μL 1× IB1 were added to each well of the ACE2-coated plate. Then, 10 μL of inhibitor solution or inhibitor buffer were mixed with 20 μL of SARS-CoV-2 Spike S1-Biotin solution in a tube and incubated at room temperature for 30 min under slow shaking. These solutions were added to the samples (with inhibitors) or the “Positive Control” (without inhibitors) wells. In turn, a mixture of 10 μL inhibitor buffer and 20 μL of 1× IB1 was added to the “Blank” wells. Incubations at room temperature for 1 h under slow shaking were performed. In Treatment C, 20 μL 1× IB1 were added to each well of the ACE2-coated plate, followed by 20 μL of SARS-CoV-2 Spike S1-Biotin solution for the samples and positive wells, and 20 μL of only 1× IB1 for the “Blank” wells, incubating at room temperature for 30 min under slow shaking. Then, 10 μL of inhibitor solutions were added to the sample wells, whereas 10 μL of the inhibitor buffer were used for the “Positive Control” and “Blank” wells. Incubation at room temperature for 1 h under slow shaking were performed.

The final DMSO concentration in the assays was 1%.

After each protein incubation, a washing-blocking-washing cycle was performed on each well; washings were carried out with 1× IB1 (3 × 100 µL per well), whereas blocking was performed incubating the well with BB2 for 10 min.

Finally, Streptavidin-HRP was immobilized on the plate and the chemiluminescence was recorded. In detail, 100 μL of diluted Streptavidin-HRP were added to each well and incubated for 1 h at room temperature under gentle shaking. After blocking the plate with BB2 for 10 min and removing the surnatant, ECL-A substrate solution was mixed with ECL-B (1:1, *v*/*v*) in a tube and 100 μL of this solution were added to each well. Chemiluminescence was immediately read in a luminometer (integration time is 1s, delay after plate movement is 100 ms; light emission reading is with no filter).

All samples and blanks were tested in duplicate, positive controls in triplicate.

The optimization of the protocol for the Spike-ACE2 binding assays was performed at two different temperatures: 10 °C and room temperature (22 °C).

## 3. Results and Discussion

### 3.1. Molecular Docking Simulations

#### 3.1.1. Binding to SARS-CoV-2 Spike Protein

Spike-protein pre-fusion conformation [50,76] is a trimer constituted of two subunits, S1 and S2, which are cleaved following receptor binding [77]. S1 Receptor Binding Domains (RBDs) host the binding motifs (RBMs) able to recognize ACE2. The high RBD flexibility allows the Spike to sample open or closed conformations, in which RBMs are respectively exposed or hidden inside the protomers interface [77,78,79,80,81]. Therefore, to assess PD and RESV inhibition capabilities of the Spike/ACE2 recognition, the binding to SARS-CoV-2 Spike structure with one RBD in an open conformation (PDB ID: 6VSB [50]) has been investigated.

Molecular docking simulations were performed onto both the A chain RBD surface and the exposed A/B and A/C interfaces at the base of A-RBD (please, refer to Figure S2 in the Supporting Information).

**Figure 2:**
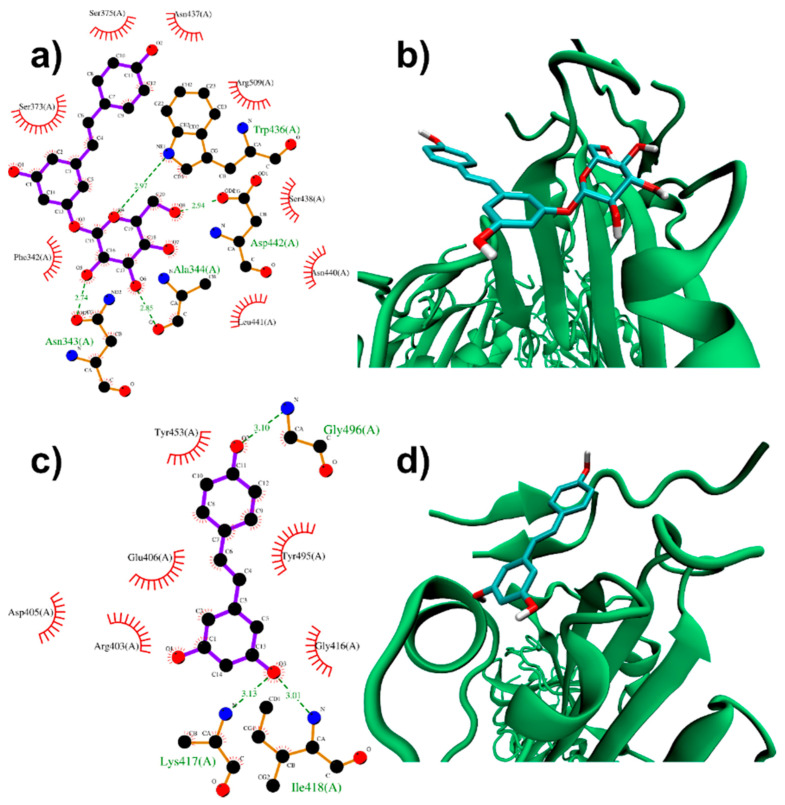
PD (**a**,**b**) and RESV (**c**,**d**) top-ranked poses docked to Spike RBD. Two-dimensional interaction maps (**a**–**c**): C, N and O atoms are reported in black, blue and red, respectively. This colour code is adopted also in the following two-dimensional interactions maps. Hydrogen bonds are depicted as green dashed lines, while hydrophobic interactions as red cogwheels. Hydrogen atoms are not depicted for ease of illustration. The names of protein residues involved in interactions with the ligand are reported. Three-dimensional representations of PD and RESV docked poses (**b**–**d**): Spike backbone is represented as a cartoon, while the ligand as sticks.

The pockets on the RBD surface appear able to accommodate both PD and RESV ligands. A closer inspection of PD top-ranked pose (−6.9 kcal/mol score, please refer to Table 1 for the docking scores of all top-ranked docked poses and Table S2 for the complete outputs of the docking simulations) shows that the PD glucose residue establishes hydrogen bonds interactions with Asn343, Ala344, Asp442 and Trp436 (Figure 2a,b, see also Table S1 for a complete list of residues composing the binding pockets). The stilbene moiety of PD is in turn involved in hydrophobic interactions with the surrounding residues. The glucose residue appears therefore relevant for PD binding capabilities, allowing several interactions through its OH groups. At the same time, the glycosidic bond torsional flexibility contributes to better fit the exposed RBD pockets.

In contrast to PD, a −6.5 kcal/mol binding mode was found for RESV (Figure 2c,d), suggesting a slightly lower affinity of this ligand for the RBD domain. This docking pose is localized on top of the open RBD domain, close to the residues interacting with ACE2. In detail, the resveratrol OH groups take part in H-bonds (with Lys417, Ile418 and Gly496). Hydrophobic contacts (such as those with Tyr453, Tyr495 and Gly416) also contribute to the binding.

These binding modes are quite close to Lys417, Tyr453, Gln498, Thr500, Gln474, Phe486 and Asn501, which interact with the ACE2 protease domain residues in the ACE2:Spike complex [51]. Molecular docking simulations, therefore, suggest a potential capability of PD and RESV to directly interfere with the Spike/ACE2 interaction. Of course, more sophisticated computational approaches (such as Molecular Dynamics simulations) are required to test this hypothesis.

The interface regions between the A and B-C chains near the exposed RBD domain (more distant from the residues directly involved in the ACE2 interaction) are characterized by pockets potentially able to accommodate ligands. In fact, PD shows −7.3 kcal/mol (Table 1) binding modes at the basis of A chain RBD (Figure S3). Looking in detail at these binding modes, glucose moiety participates in H-bonds with Asn394 (A), Ser359 (A), Asn360 (A), Pro230 (B), Ile231(B) in the A/B interface docked pose (Figure S3a,b) and with Phe377 (A), Cys379 (A) in the A/C interface pose, where a resveratrol moiety OH group also binds to Phe490 (C) (Figure S3c,d).

Interestingly, only >−6.5 kcal/mol docked poses were found for RESV at these interface regions, appearing therefore highly selective for PD, because of the interaction capabilities of the glucose moiety.

#### 3.1.2. Binding to ACE2 Receptor

ACE2 homodimer (PDB ID: 6M18, [51] Figure S4) is constituted by an N-terminal protease domain and a C-terminal collectrin-like domain, comprising the ACE2 transmembrane helix. In particular, protease domain Gln24, Asp30, His34, Tyr41, Gln42 (α1 helix), Met82 (α2 helix), Lys353 and Arg357 (β3/β4 loop) residues are involved in the interaction with SARS-CoV-2 Spike RBD [51].

**Figure 3:**
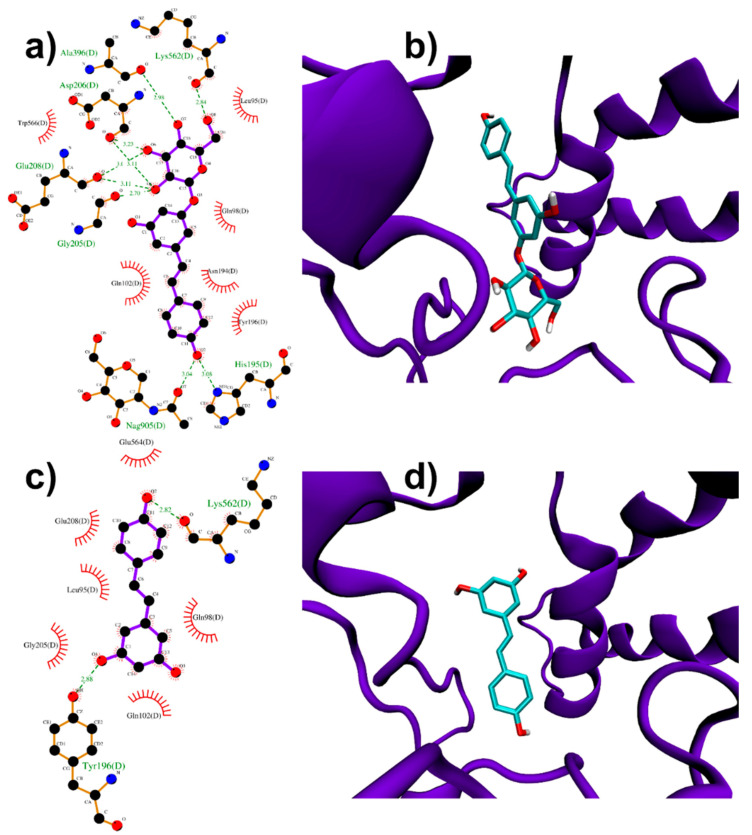
PD (**a**,**b**) and RESV (**c**,**d**) top-ranked poses docked to ACE2.

Molecular docking simulations were performed near ACE2 protease domain α1 and α2 helices, thus far from the catalytic site related to ACE2 physiological function. Despite its size, PD can be easily accommodated in a deep groove behind the two helices (Figure 3a,b). In particular, the high docking score (−8.4 kcal/mol, Table 1) is produced by several polar glucose (with Gly205, Glu208, Asp206, Ala396, Lys562) and resveratrol moiety (with N-acetylglucosamine 905, His195) interactions.

RESV can also bind to the pockets near α1 and α2 helices, but with a ~1 kcal/mol lower score with respect to PD (−6.9 kcal/mol, Table 1). Two polar interactions by resveratrol OH groups (with Tyr196 and Lys562) are found (Figure 3c,d).

These results from Molecular Docking simulations (particularly those for PD) suggest only the possibility that these ligands bound near ACE2 interaction region with viral Spike could exert some direct (steric) or indirect (allosteric) effect in the recognition process, justifying future efforts, e.g., Molecular Dynamics investigations, to further corroborate this hypothesis.

#### 3.1.3. Binding to ACE2:Spike Complex

Both RESV and PD showed a good estimated binding affinity in the groove between the Spike RBD and ACE2 (see Figure S5 for a three-dimensional representation of the Spike:ACE2 complex). Due to the high spatial extent of the Spike:ACE2 interface, docking simulations were performed in two distinct regions (named regions I and II, Figure S6).

**Figure 4:**
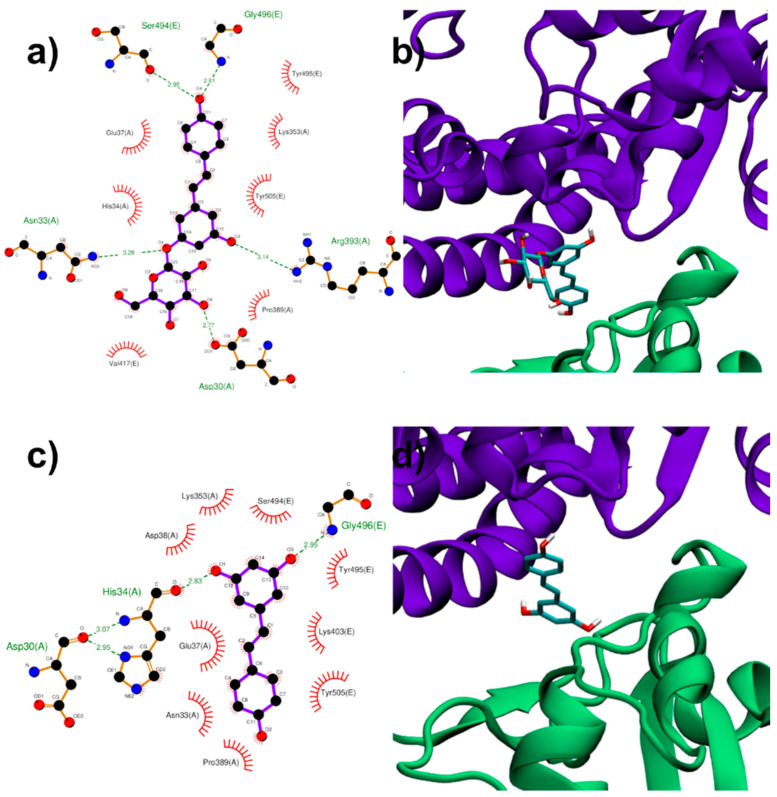
PD (**a**,**b**) and RESV (**c**,**d**) top-ranked poses docked to Spike:ACE2 interface region I.

In the first interfacial region investigated (region I), the most stable RESV binding mode involves recognition of one of the interfacial pockets through polar and hydrophobic contacts (Figure 4c,d). The planar stilbenoid structure slips almost parallel to the two different chains, in contact with the viral Spike RBD on one side, stabilized by hydrophobic interactions with Ser494, Tyr495, Lys403 and Tyr505, and similarly interacting on the other side with the proximal residues of ACE2 (Lys353, Glu37, Asp38, Asn33 and Pro389). In turn, the aromatic ring bearing the two hydroxyl groups strengthens the interaction by engaging two strong hydrogen bonds with Gly496 (N(Gly496)-O3 2.95 Å), located on an external loop of the Spike protein, and His34 (O(His34)-O1 2.83 Å) belonging to an ACE2 α-helix.

The hydroxyl groups of the glycosidic part make PD more prone to polar interactions with the host with respect to the aglycone counterpart. PD binds in the same pocket as RESV but interacting with more surrounding residues, thus showing a higher docking score (−8.1 kcal/mol, Table 1), compared to that (−7.6 kcal/mol) of RESV.

The stilbenoid scaffold reaches a similar position as in the case of RESV but is now reversed, and the glucose unit, being free to rotate, places orthogonal to it, pointing mainly towards the viral Spike RBD (Figure 4a,b). PD shares most hydrophobic contacts of RESV on both sides, engaging five hydrogen bonds, three with ACE2 residues as Asp30, Asn33 and Arg393 (O(Asp30)-O6 2.77 Å, N (Asn33)-O1 3.28 Å and N (Arg393)-O3 3.14 Å), while two Spike residues, Ser494 and Gly496, bind the same oxygen atom from the opposite side of the molecule.

In the second region (region II, Figure S6) investigated at the interface between viral Spike RBD and human ACE2, the top-ranked RESV conformer fits into a large ACE2 cavity far from the interaction sites between the two protein domains. The disubstituted ring faces a turn and an α-helix, the ethylene bridge lies along a β-sheet while the other benzene ring interacts with the α-helix more involved in the interaction with the Spike RBD (Figure S7c,d). In this pocket, the ligand engages only two hydrogen bonds with two different hydroxyl groups, with Asp382 (O(Asp382)-O1 2.71 Å) on one side and Ser43 (O(Ser43)-O2 3.19 Å) on the other side. The binding is reinforced by hydrophobic contacts with eight surrounding residues as shown as red cogwheels in Figure S7c.

On the contrary, the best PD binding mode (Figure S7a,b) binds onto a pocket located at one end of the ACE2:Spike complex interface with a docking score 0.4 kcal/mol lower than RESV (−6.9 kcal/mol, Table 1). In detail, the aromatic rings of the stilbenic portion interact hydrophobically mainly with side chains of ACE2 residues (Thr324, Gly354 and Ala387), while the perpendicularly arranged glucose unit engages multiple hydrogen bonds with polar residues of the viral Spike. It is interesting to note the fundamental role played by the glucosidic moiety which interacts with the residues of the main chains that are involved in the formation of the ACE2:Spike dimer, such as Gly502 (E), Tyr505 (E), Gly354 (A) and Arg408 (E), see Figure S7a. Weakening of these interactions could, in principle, contribute to the dissociation of the dimeric complex.

Results from docking simulations on the already assembled Spike:ACE2 complex reveal the potential capability of PD, but also RESV, to insert themselves into the extended adduct interface. This leads to the hypothesis of a ligand-induced dissociation or weakening effect, through allosteric (such as for region I) or direct (region II) interference. Again, it must be stressed that further computational investigations (e.g., Molecular Dynamics) are required to provide direct evidence.

Our results about RESV docking on the Spike:ACE2 interface (location of the docked pose and docking score) largely agree with those in ref. [38], further validating our Molecular Docking protocol.

### 3.2. Preliminary ACE2:Spike Binding Inhibition Assays

To establish if RESV and PD can experimentally interfere with the binding of the Spike protein with ACE2 receptor, as suggested by the molecular docking simulations, binding inhibition assays were performed.

The assay we carried out was based on the following steps: (1) immobilization of the purified ACE2 protein, labelled with a His-tag (ACE2-His), on a Ni-coated 96-well plate; (2) attachment of biotinylated SARS-CoV-2 Spike S1 protein (from here on named just Spike) on the ACE2-functionalized plate, exploiting the high affinity of Spike for ACE2; (3) binding of streptavidin-horseradish peroxidase (HRP) to the bound Spike, thanks to the high recognition affinity between biotin and streptavidin; (4) treatment of the so-prepared plate with an HRP-substrate to produce chemiluminescence, measured at the end of the assay using a luminescence reader. Chemiluminescence intensity is correlated with the amount of Spike attached to the plate. If an inhibitor of the ACE2:Spike interaction is added to the plate, a reduction in chemiluminescence can be observed, proportional to the efficacy of the inhibition. Chemiluminescence reading in wells treated in the same way as the samples but without Spike and inhibitors (RESV/PD) were called “Blanks”, whereas those without inhibitors were named “Positive controls”. The chemiluminescence reduction observed in the wells treated with PD and RESV was converted in % inhibition of the ACE2:Spike binding by the stilbenoids; this was obtained by subtracting the chemiluminescence value of the blank from that of all the other treatments and calculating the complementary percentage relative to the positive control.

We first explored the concentration of RESV and PD which could efficiently inhibit the ACE2:Spike interaction. Following the protocol suggested in the datasheet of the used assay kit, we pre-incubated RESV or PD at 40 μM, for 30 min, with the ACE2-functionalized plate, before adding Spike-biotin. Under these conditions, we did not observe any significant effect in two independent experiments (Figure S8).

We thus decided to optimize the assay protocol using a higher concentration of the two polyphenols (250 μM). We explored three different treatments: (1) Treatment A, i.e., the protocol suggested in the datasheet of the used assay kit, with a pre-incubation of RESV/PD with ACE2-coated plate; (2) Treatment B, involving a pre-incubation of RESV/PD with Spike S1 in solution; (3) Treatment C, involving a pre-incubation of Spike S1 with ACE2-coated plate.

**Figure 5:**
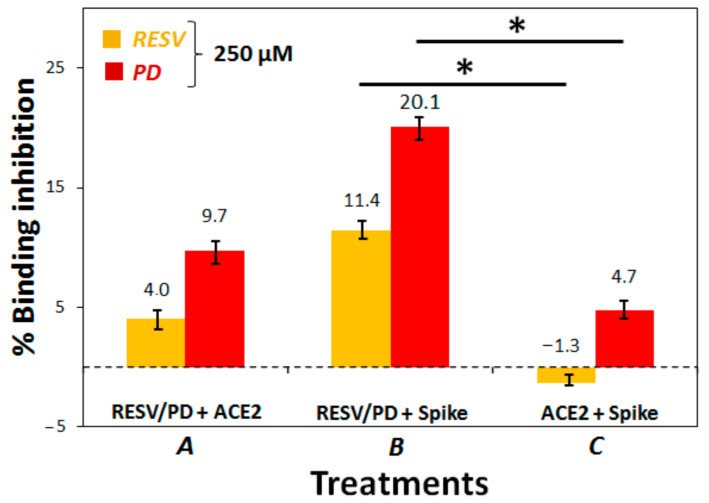
ACE2:Spike inhibition binding assay. In Treatment A, the polyphenols were pre-incubated with ACE2 on the plate, and then Spike was added; in Treatment B, the polyphenols were pre-incubated with Spike in solution, and this mixture was then added to ACE2 on the plate; in Treatment C, ACE2 and Spike were pre-incubated on the plate and then the polyphenols were added. Percentages of binding inhibition were calculated correlating the chemiluminescence intensity readings (Figure S9) to that of the positive control. *p*-Values have been calculated using the Student’s *t*-test (* *p* ≤ 0.05).

In all cases, we noted that RESV always produced a smaller effect than PD. This observation is consistent with the Molecular Docking results, always showing a higher docking score of PD for all the investigated targets (Table 1). Moreover, we evidenced that the more convenient treatment, among the three, explored ones, was B, consisting of the pre-incubation of RESV/PD with Spike in solution before addition of ACE2 (Figure 5 and Figure S9 for the chemiluminescence data). In particular, the percentage of ACE2:Spike binding-inhibition produced by PD, in this case, was about 20%.

Treatments A and B were also repeated at 200 μM concentration of the two natural compounds confirming the observed trend (Figure S10).

**Figure 6:**
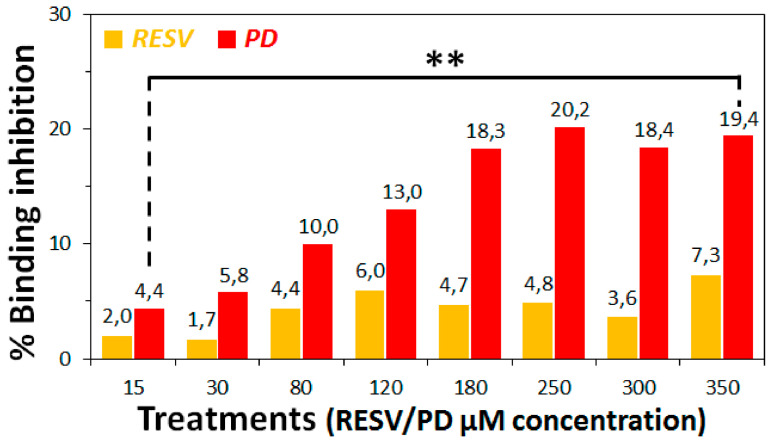
ACE2:Spike inhibition binding assay. In all treatments, the polyphenols were pre-incubated with Spike in solution. Chemiluminescence intensities were measured on the 96-well plate with a luminescence reader and converted in percentages of ACE2:Spike-binding inhibition with respect to the positive control. *p*-values have been calculated using the Student’s *t*-test (** *p* ≤ 0.01).

Subsequently, a range of suitable concentrations (0–350 μM) of RESV and PD were explored for the ACE2:Spike-binding inhibition assay under the optimal conditions found (Treatment B). This experiment afforded the chemiluminescence data reported in Figure S11a, evidencing a dose-response effect in the case of PD treatments in the range 0–250 μM, while higher concentrations did not produce additional effects. RESV, in our hands, did not seem to afford significant effects. Conversion of the chemiluminescence data in percentages of ACE2:Spike binding-inhibition by RESV and PD was reported in Figure 6 and Figure S11b.

Analysis of these data evidenced that the highest effect was obtained at 250 μM PD concentration, with a binding-inhibitory activity of ca. 20%.

Thus, in the conditions of this specific assay, we could not calculate the IC_50_ value for PD since we did not reach the 100% binding inhibition. This behaviour could be probably due to solubility and aggregation issues of the two polyphenols, especially RESV [82,83], in the assay buffer conditions.

The assays of Figure 6, as well as those of Figure 5, were performed also at 10 °C without evidencing any significant difference on varying the temperature (data not shown), overall confirming the observed trend.

These preliminary experimental assays directly revealed a PD inhibitory action of the ACE2:Spike interaction, in agreement with the Molecular Docking simulations on the surface regions of ACE2, Spike and their complex (corresponding to the experimental conditions here named Treatments A, B and C), demonstrating some binding capabilities by PD. RESV in turn did not produce a significant binding inhibition under the assay conditions. This could be mainly due to solubility and aggregation issues of RESV, which are more critical than for PD. In addition, even if the binding of RESV occurs, this could not impede the interaction between ACE2 and Spike proteins. Indeed docking simulations predicted a lower binding score by RESV for both isolated Spike and ACE2 and their complex.

## 4. Conclusions

In this work, the binding abilities of the natural compounds polydatin (PD) and resveratrol (RESV) towards two key targets involved in SARS-CoV-2 viral infection—Spike viral protein and ACE2 host receptor—were investigated by molecular docking simulations.

In particular, we here studied the interactions of PD/RESV with both Spike and ACE2 as separated proteins, as well as with their complex, through a molecular docking-based computational approach, using the PDB available molecular structures.

Molecular docking targeted at Spike and ACE2 surface pockets near their interaction sites and the interface of the already assembled ACE2:Spike complex revealed potential binding and insertion capabilities by both PD and RESV ligands. In all cases, the predicted binding with PD appeared stronger than with RESV. These Molecular Docking data thus encourage further computational investigations aimed at verifying PD and RESV interference or weakening effects in the ACE2:Spike recognition.

Furthermore, aiming at supporting the data obtained from molecular docking simulations, preliminary biochemical assays were performed to experimentally evaluate the ability of PD/RESV to interfere with the binding of the Spike protein with the ACE2 receptor. Our assays evidenced a dose-response effect in the case of PD reaching a maximum of >20% ACE2:Spike binding inhibition at 250 μM PD concentration.

Even if high concentrations were required to obtain a significant effect in this kind of experiment, we were encouraged from the obtained results due to the known absence of side effects and toxicity of PD even at high dosage, as demonstrated by its use as a nutraceutical product (as a human food supplement, the recommended dose of polydatin is 160 mg/day for assumption cycles of at least three months [84]) and in clinical applications [85,86].

In addition, we have here showed a biochemical assay not considering (i) several biological aspects of ACE2-Spike binding only identifiable by cellular assays, e.g., the role of biological multimerization [51], (ii) solubility issues and aggregation state of the studied polyphenols, especially RESV [82,83], in the assay buffer conditions (not considered by the modelling studies), (iii) synergistic effects deriving from the interaction of these polyphenols with other key viral proteins or other host targets, which could reinforce the overall result.

From the current picture, PD emerges as a potential candidate drug/protective agent, which can act as a sort of “biological mask”. It can inhibit the binding of Spike to ACE2 and therefore reduce viral entry into host cells, also being well-known its favourable properties like high water solubility and metabolic stability, good oral absorption and absence of side effects, as well as beneficial and protective effects during inflammation particularly of the respiratory tract [87].

## Figures and Tables

**Table 1:** Docking scores (i.e., the approximate binding energy estimated by the docking scoring function, kcal/mol units) for PD and RESV top-ranked docked poses.

	PD	RESV
Spike RBD	−6.9	−6.5
Spike A/B interface	−7.3	>−6.5
Spike A/C interface	−7.3	>−6.5
ACE2	−8.4	−6.9
S:ACE2 region I	−8.1	−7.6
S:ACE2 region II	−6.9	−6.5

## Data Availability

Not applicable.

## Supplementary Materials

The following are available online at https://www.mdpi.com/article/10.3390/biom11071048/s1, Figure S1: Structures of trans-resveratrol and trans-polydatin ligands in ball and stick representation; Figure S2: SARS-CoV-2 Spike protein with one RBD in an open conformation (PDB ID: 6VSB); Figure S3: PD top-ranked poses docked to Spike A-chain RBD/B and A-chain RBD/C interfaces; Figure S4: Human ACE2 receptor (PDB ID: 6M18); Figure S5: Three-dimensional structure of the SARS-CoV-2 chimeric receptor-binding domain complexed with its receptor human ACE2 (PDB ID: 6VW1); Figure S6: Cubic grids used in molecular docking simulations centred on two distinct interfacial regions of the Spike:ACE2 complex; Figure S7: PD and RESV top-ranked poses docked to Spike:ACE2 interface region II; Table S1: Description of the binding pocket for each PD and RESV docked pose; Table S2: output of docking simulations; Figure S8: ACE2:Spike Inhibition binding assay (40 µM); Figure S9: ACE2:Spike inhibition binding assay (luminescence, 250 µM); Figure S10: ACE2:Spike Inhibition binding assay (200 µM); Figure S11: ACE2:Spike Inhibition binding assay (15–350 µM).

## Abbreviations

Angiotensin-converting enzyme (ACE2), Binding energy (BE), Blocking buffer 2 (BB2), Coronavirus (CoV), Coronavirus disease 2019 (COVID-19), Cryogenic electron microscopy (Cryo-EM), Density functional theory (DFT), Dimethyl Sulfoxide (DMSO), Enhanced chemiluminescence (ECL), Envelope (E), Enzyme-linked immunosorbent assay (ELISA), Horseradish peroxidase (HRP), Immuno buffer 1 (IB1), Membrane (M) Nucleocapsid (N), Phosphate-buffered saline (PBS), Protein Data Bank (PDB), Receptor-binding domain (RBD), Receptor binding motif (RBM), Spike (S), trans-polydatin (PD), trans-resveratrol (RESV), Type II transmembrane serine protease (TMPRSS2).

## References

[1] Fehr A.R., Perlman S. (2015). Coronaviruses: An Overview of Their Replication and Pathogenesis. Coronaviruses: Methods and Protocols.

[2] Masters P.S. (2006). The Molecular Biology of Coronaviruses. Adv. Virus Res..

[3] Corman V.M., Lienau J., Witzenrath M. (2019). Coronaviruses as the Cause of Respiratory Infections. Internist.

[4] Huang C., Wang Y., Li X., Ren L., Zhao J., Hu Y., Zhang L., Fan G., Xu J., Gu X. (2020). Clinical Features of Patients Infected with 2019 Novel Coronavirus in Wuhan, China. Lancet.

[5] Wu F., Zhao S., Yu B., Chen Y.M., Wang W., Song Z.G., Hu Y., Tao Z.W., Tian J.H., Pei Y.Y. (2020). A New Coronavirus Associated with Human Respiratory Disease in China. Nature.

[6] Zhou P., Yang X.-L., Wang X.-G., Hu B., Zhang L., Zhang W., Si H.-R., Zhu Y., Li B., Huang C.-L. (2020). A Pneumonia Outbreak Associated with a New Coronavirus of Probable Bat Origin. Nature.

[7] Siu Y.L., Teoh K.T., Lo J., Chan C.M., Kien F., Escriou N., Tsao S.W., Nicholls J.M., Altmeyer R., Peiris J.S.M. (2008). The M, E, and N Structural Proteins of the Severe Acute Respiratory Syndrome Coronavirus Are Required for Efficient Assembly, Trafficking, and Release of Virus-Like Particles. J. Virol..

[8] Belouzard S., Millet J.K., Licitra B.N., Whittaker G.R. (2012). Mechanisms of Coronavirus Cell Entry Mediated by the Viral Spike Protein. Viruses.

[9] Shang J., Wan Y., Luo C., Ye G., Geng Q., Auerbach A., Li F. (2020). Cell Entry Mechanisms of SARS-CoV-2. Proc. Natl. Acad. Sci. USA.

[10] Shang J., Ye G., Shi K., Wan Y., Luo C., Aihara H., Geng Q., Auerbach A., Li F. (2020). Structural Basis of Receptor Recognition by SARS-CoV-2. Nature.

[11] Wan Y., Shang J., Graham R., Baric R.S., Li F. (2020). Receptor Recognition by the Novel Coronavirus from Wuhan: An Analysis Based on Decade-Long Structural Studies of SARS Coronavirus. J. Virol..

[12] Bourgonje A.R., Abdulle A.E., Timens W., Hillebrands J.L., Navis G.J., Gordijn S.J., Bolling M.C., Dijkstra G., Voors A.A., Osterhaus A.D.M.E. (2020). Angiotensin-Converting Enzyme 2 (ACE2), SARS-CoV-2 and the Pathophysiology of Coronavirus Disease 2019 (COVID-19). J. Pathol..

[13] Lukassen S., Chua R.L., Trefzer T., Kahn N.C., Schneider M.A., Muley T., Winter H., Meister M., Veith C., Boots A.W. (2020). SARS-CoV-2 Receptor ACE 2 and TMPRSS 2 Are Primarily Expressed in Bronchial Transient Secretory Cells. EMBO J..

[14] Magrone T., Magrone M., Jirillo E. (2020). Focus on Receptors for Coronaviruses with Special Reference to Angiotensin- Converting Enzyme 2 as a Potential Drug Target—A Perspective. Endocr. Metab. Immune Disord. Drug Targets.

[15] Chi X., Yan R., Zhang J., Zhang G., Zhang Y., Hao M., Zhang Z., Fan P., Dong Y., Yang Y. (2020). A Neutralizing Human Antibody Binds to the N-Terminal Domain of the Spike Protein of SARS-CoV-2. Science.

[16] Liu L., Wang P., Nair M.S., Yu J., Rapp M., Wang Q., Luo Y., Chan J.F.W., Sahi V., Figueroa A. (2020). Potent Neutralizing Antibodies against Multiple Epitopes on SARS-CoV-2 Spike. Nature.

[17] Papageorgiou A.C., Mohsin I. (2020). The SARS-CoV-2 Spike Glycoprotein as a Drug and Vaccine Target: Structural Insights into Its Complexes with ACE2 and Antibodies. Cells.

[18] Yi C., Sun X., Ye J., Ding L., Liu M., Yang Z., Lu X., Zhang Y., Ma L., Gu W. (2020). Key Residues of the Receptor Binding Motif in the Spike Protein of SARS-CoV-2 That Interact with ACE2 and Neutralizing Antibodies. Cell. Mol. Immunol..

[19] Ju B., Zhang Q., Ge J., Wang R., Sun J., Ge X., Yu J., Shan S., Zhou B., Song S. (2020). Human Neutralizing Antibodies Elicited by SARS-CoV-2 Infection. Nature.

[20] Hoffmann M., Kleine-Weber H., Schroeder S., Krüger N., Herrler T., Erichsen S., Schiergens T.S., Herrler G., Wu N.H., Nitsche A. (2020). SARS-CoV-2 Cell Entry Depends on ACE2 and TMPRSS2 and Is Blocked by a Clinically Proven Protease Inhibitor. Cell.

[21] Gil C., Ginex T., Maestro I., Nozal V., Barrado-Gil L., Cuesta-Geijo M.Á., Urquiza J., Ramírez D., Alonso C., Campillo N.E. (2020). COVID-19: Drug Targets and Potential Treatments. J. Med. Chem..

[22] Hensel A., Bauer R., Heinrich M., Spiegler V., Kayser O., Hempel G., Kraft K. (2020). Challenges at the Time of COVID-19: Opportunities and Innovations in Antivirals from Nature. Planta Med..

[23] Islam M.T., Sarkar C., El-Kersh D.M., Jamaddar S., Uddin S.J., Shilpi J.A., Mubarak M.S. (2020). Natural Products and Their Derivatives against Coronavirus: A Review of the Non-clinical and Pre-clinical Data. Phyther. Res..

[24] Mani J.S., Johnson J.B., Steel J.C., Broszczak D.A., Neilsen P.M., Walsh K.B., Naiker M. (2020). Natural Product-Derived Phytochemicals as Potential Agents against Coronaviruses: A Review. Virus Res..

[25] Romeo I., Mesiti F., Lupia A., Alcaro S. (2021). Current Updates on Naturally Occurring Compounds Recognizing SARS-CoV-2 Druggable Targets. Molecules.

[26] Quideau S., Deffieux D., Douat-Casassus C., Pouységu L. (2011). Plant Polyphenols: Chemical Properties, Biological Activities, and Synthesis. Angew. Chemie Int. Ed..

[27] Gambini J., Inglés M., Olaso G., Lopez-Grueso R., Bonet-Costa V., Gimeno-Mallench L., Mas-Bargues C., Abdelaziz K.M., Gomez-Cabrera M.C., Vina J. (2015). Properties of Resveratrol: In Vitro and In Vivo Studies about Metabolism, Bioavailability, and Biological Effects in Animal Models and Humans. Oxid. Med. Cell. Longev..

[28] Du Q.H., Peng C., Zhang H. (2013). Polydatin: A Review of Pharmacology and Pharmacokinetics. Pharm. Biol..

[29] Zorzete P., Reis T.A., Felício J.D., Baquião A.C., Makimoto P., Corrêa B. (2011). Fungi, Mycotoxins and Phytoalexin in Peanut Varieties, during Plant Growth in the Field. Food Chem..

[30] Regev-Shoshani G., Shoseyov O., Bilkis I., Kerem Z. (2003). Glycosylation of Resveratrol Protects It from Enzymic Oxidation. Biochem. J..

[31] Fabris S., Momo F., Ravagnan G., Stevanato R. (2008). Antioxidant Properties of Resveratrol and Piceid on Lipid Peroxidation in Micelles and Monolamellar Liposomes. Biophys. Chem..

[32] Platella C., Guida S., Bonmassar L., Aquino A., Bonmassar E., Ravagnan G., Montesarchio D., Roviello G.N., Musumeci D., Fuggetta M.P. (2017). Antitumour Activity of Resveratrol on Human Melanoma Cells: A Possible Mechanism Related to Its Interaction with Malignant Cell Telomerase. Biochim. Biophys. Acta Gen. Subj..

[33] Platella C., Raucci U., Rega N., D’Atri S., Levati L., Roviello G.N., Fuggetta M.P., Musumeci D., Montesarchio D. (2020). Shedding Light on the Interaction of Polydatin and Resveratrol with G-Quadruplex and Duplex DNA: A Biophysical, Computational and Biological Approach. Int. J. Biol. Macromol..

[34] Francioso A., Mastromarino P., Masci A., D’Erme M., Mosca L. (2014). Chemistry, Stability and Bioavailability of Resveratrol. Med. Chem..

[35] Şöhretoğlu D., Baran M.Y., Arroo R., Kuruüzüm-Uz A. (2018). Recent Advances in Chemistry, Therapeutic Properties and Sources of Polydatin. Phytochem. Rev..

[36] Magrone T., Magrone M., Russo M.A., Jirillo E. (2019). Recent Advances on the Anti-Inflammatory and Antioxidant Properties of Red Grape Polyphenols: In Vitro and In Vivo Studies. Antioxidants.

[37] Dong L., Hu S., Gao J. (2020). Discovering Drugs to Treat Coronavirus Disease 2019 (COVID-19). Drug Discov. Ther..

[38] Wahedi H.M., Ahmad S., Abbasi S.W. (2020). Stilbene-Based Natural Compounds as Promising Drug Candidates against COVID-19. J. Biomol. Struct. Dyn..

[39] Yang M., Wei J., Huang T., Lei L., Shen C., Lai J., Yang M., Liu L., Yang Y., Liu G. (2020). Resveratrol Inhibits the Replication of Severe Acute Respiratory Syndrome Coronavirus 2 (SARS-CoV-2) in Cultured Vero Cells. Phyther. Res..

[40] Feng H., Choi H.Y., Cui C., Xu H., Jiang J., Yan G., Jin M. (2018). Effects of Polydatin on Oleic Acid-Induced Acute Respiratory Distress Syndrome in Rats. Int. J. Clin. Exp. Med..

[41] Filardo S., Di Pietro M., Mastromarino P., Sessa R. (2020). Therapeutic Potential of Resveratrol against Emerging Respiratory Viral Infections. Pharmacol. Ther..

[42] Jiang Q., Yi M., Guo Q., Wang C., Wang H., Meng S., Liu C., Fu Y., Ji H., Chen T. (2015). Protective Effects of Polydatin on Lipopolysaccharide-Induced Acute Lung Injury through TLR4-MyD88-NF-ΚB Pathway. Int. Immunopharmacol..

[43] Khalil A., Tazeddinova D. (2020). The Upshot of Polyphenolic Compounds on Immunity amid COVID-19 Pandemic and Other Emerging Communicable Diseases: An Appraisal. Nat. Products Bioprospect..

[44] Li X.H., Gong X., Zhang L., Jiang R., Li H.Z., Wu M.J., Wan J.Y. (2013). Protective Effects of Polydatin on Septic Lung Injury in Mice via Upregulation of HO-1. Mediators Inflamm..

[45] Lanzilli G., Cottarelli A., Nicotera G., Guida S., Ravagnan G., Fuggetta M.P. (2012). Anti-Inflammatory Effect of Resveratrol and Polydatin by in Vitro IL-17 Modulation. Inflammation.

[46] Lo Muzio L., Bizzoca M.E., Ravagnan G. (2020). New Intriguing Possibility for Prevention of Coronavirus Pneumonitis: Natural Purified Polyphenols. Oral Dis..

[47] Yan X.-D., Wang Q.-M., Tie C., Jin H.-T., Han Y.-X., Zhang J.-L., Yu X.-M., Hou Q., Zhang P.-P., Wang A.-P. (2017). Polydatin Protects the Respiratory System from PM2.5 Exposure. Sci. Rep..

[48] Ravagnan G., De Filippis A., Cartenì M., De Maria S., Cozza V., Petrazzuolo M., Tufano M.A., Donnarumma G. (2013). Polydatin, A Natural Precursor of Resveratrol, Induces β-Defensin Production and Reduces Inflammatory Response. Inflammation.

[49] Pasquereau S., Nehme Z., Haidar Ahmad S., Daouad F., Van Assche J., Wallet C., Schwartz C., Rohr O., Morot-Bizot S., Herbein G. (2021). Resveratrol Inhibits HCoV-229E and SARS-CoV-2 Coronavirus Replication In Vitro. Viruses.

[50] Wrapp D., Wang N., Corbett K.S., Goldsmith J.A., Hsieh C.-L., Abiona O., Graham B.S., McLellan J.S. (2020). Cryo-EM Structure of the 2019-NCoV Spike in the Prefusion Conformation. Science.

[51] Yan R., Zhang Y., Li Y., Xia L., Guo Y., Zhou Q. (2020). Structural Basis for the Recognition of SARS-CoV-2 by Full-Length Human ACE2. Science.

[52] Morris G.M., Huey R., Lindstrom W., Sanner M.F., Belew R.K., Goodsell D.S., Olson A.J. (2009). AutoDock4 and AutoDockTools4: Automated Docking with Selective Receptor Flexibility. J. Comput. Chem..

[53] Dennington R., Keith T., Millam J. (2009). GaussView, Version 6.

[54] Becke A.D. (1988). Density-Functional Exchange-Energy Approximation with Correct Asymptotic Behavior. Phys. Rev. A.

[55] Lee C., Yang W., Parr R.G. (1988). Development of the Colle-Salvetti Correlation-Energy Formula into a Functional of the Electron Density. Phys. Rev. B.

[56] Frisch M.J., Trucks G.W., Schlegel H.B., Scuseria G.E., Robb M.A., Cheeseman J.R., Scalmani G., Barone V., Petersson G.A., Nakatsuji H. (2016). Gaussian16 Revision C.01.

[57] Trott O., Olson A.J. (2009). AutoDock Vina: Improving the Speed and Accuracy of Docking with a New Scoring Function, Efficient Optimization, and Multithreading. J. Comput. Chem..

[58] Schmidtke P., Le Guilloux V., Maupetit J., Tufféry P. (2010). Fpocket: Online Tools for Protein Ensemble Pocket Detection and Tracking. Nucleic Acids Res..

[59] Le Guilloux V., Schmidtke P., Tuffery P. (2009). Fpocket: An Open Source Platform for Ligand Pocket Detection. BMC Bioinform..

[60] Schmidtke P., Barril X. (2010). Understanding and Predicting Druggability. A High-Throughput Method for Detection of Drug Binding Sites. J. Med. Chem..

[61] Schmidtke P., Souaille C., Estienne F., Baurin N., Kroemer R.T. (2010). Large-Scale Comparison of Four Binding Site Detection Algorithms. J. Chem. Inf. Model..

[62] Ezzat A., Kwoh C.K. (2012). Comparison of Structure-Based Tools for the Prediction of Ligand Binding Site Residues in Apo-Structures. Procedia Comput. Sci..

[63] Windshügel B. (2019). Structural Insights into Ligand-Binding Pocket Formation in Nurr1 by Molecular Dynamics Simulations. J. Biomol. Struct. Dyn..

[64] Pradeepkiran J.A., Reddy P.H. (2019). Structure Based Design and Molecular Docking Studies for Phosphorylated Tau Inhibitors in Alzheimer’s Disease. Cells.

[65] Nguyen P.T.V., Yu H., Keller P.A. (2018). Molecular Docking Studies to Explore Potential Binding Pockets and Inhibitors for Chikungunya Virus Envelope Glycoproteins. Interdiscip. Sci. Comput. Life Sci..

[66] Jiang C.-H., Huang C.-X., Chen Y.-J., Chuang Y.-C., Huang B.-Y., Yang C.-N. (2018). Molecular Modeling for Structural Insights Concerning the Activation Mechanisms of F1174L and R1275Q Mutations on Anaplastic Lymphoma Kinase. Molecules.

[67] Llorach-Pares L., Rodriguez-Urgelles E., Nonell-Canals A., Alberch J., Avila C., Sanchez-Martinez M., Giralt A. (2020). Meridianins and Lignarenone B as Potential GSK3β Inhibitors and Inductors of Structural Neuronal Plasticity. Biomolecules.

[68] Vittorio S., Seidel T., Garon A., Gitto R., Langer T., De Luca L. (2021). In Silico Identification of Potential Druggable Binding Sites on CIN85 SH3 Domain. Int. J. Mol. Sci..

[69] Hetényi C., van der Spoel D. (2006). Blind Docking of Drug-Sized Compounds to Proteins with up to a Thousand Residues. FEBS Lett..

[70] Hassan N.M., Alhossary A.A., Mu Y., Kwoh C.-K. (2017). Protein-Ligand Blind Docking Using QuickVina-W with Inter-Process Spatio-Temporal Integration. Sci. Rep..

[71] Ghersi D., Sanchez R. (2009). Improving Accuracy and Efficiency of Blind Protein-Ligand Docking by Focusing on Predicted Binding Sites. Proteins Struct. Funct. Bioinform..

[72] Jofily P., Pascutti P.G., Torres P.H.M. (2021). Improving Blind Docking in DOCK6 through an Automated Preliminary Fragment Probing Strategy. Molecules.

[73] Zhou M., Luo H., Li R., Ding Z. (2013). Exploring the Binding Mode of HIV-1 Vif Inhibitors by Blind Docking, Molecular Dynamics and MM/GBSA. RSC Adv..

[74] Iorga B., Herlem D., Barré E., Guillou C. (2006). Acetylcholine Nicotinic Receptors: Finding the Putative Binding Site of Allosteric Modulators Using the “Blind Docking” Approach. J. Mol. Model..

[75] Laskowski R.A., Swindells M.B. (2011). LigPlot+: Multiple Ligand-Protein Interaction Diagrams for Drug Discovery. J. Chem. Inf. Model..

[76] Walls A.C., Park Y.-J., Tortorici M.A., Wall A., McGuire A.T., Veesler D. (2020). Structure, Function, and Antigenicity of the SARS-CoV-2 Spike Glycoprotein. Cell.

[77] Walls A.C., Tortorici M.A., Snijder J., Xiong X., Bosch B.-J., Rey F.A., Veesler D. (2017). Tectonic Conformational Changes of a Coronavirus Spike Glycoprotein Promote Membrane Fusion. Proc. Natl. Acad. Sci. USA.

[78] Song W., Gui M., Wang X., Xiang Y. (2018). Cryo-EM Structure of the SARS Coronavirus Spike Glycoprotein in Complex with Its Host Cell Receptor ACE2. PLoS Pathog..

[79] Walls A.C., Xiong X., Park Y.-J., Tortorici M.A., Snijder J., Quispe J., Cameroni E., Gopal R., Dai M., Lanzavecchia A. (2019). Unexpected Receptor Functional Mimicry Elucidates Activation of Coronavirus Fusion. Cell.

[80] Li Z., Tomlinson A.C.A., Wong A.H.M., Zhou D., Desforges M., Talbot P.J., Benlekbir S., Rubinstein J.L., Rini J.M. (2019). The Human Coronavirus HCoV-229E S-Protein Structure and Receptor Binding. eLife.

[81] Walls A.C., Tortorici M.A., Frenz B., Snijder J., Li W., Rey F.A., DiMaio F., Bosch B.-J., Veesler D. (2016). Glycan Shield and Epitope Masking of a Coronavirus Spike Protein Observed by Cryo-Electron Microscopy. Nat. Struct. Mol. Biol..

[82] López-Nicolás J.M., Pérez-Gilabert M., García-Carmona F. (2009). Effect of Protonation and Aggregation State of (E)-Resveratrol on Its Hydroperoxidation by Lipoxygenase. J. Agric. Food Chem..

[83] López-Nicolás J.M., García-Carmona F. (2008). Aggregation State and PKa Values of (E)-Resveratrol As Determined by Fluorescence Spectroscopy and UV−Visible Absorption. J. Agric. Food Chem..

[84] Polidase^®^, the Bioavailable Resveratrol, Sherman Tree Nutraceuticals. https://shermantree.it/en/polidase/.

[85] Izzo G.M., Suffritti G. (2017). Polydatin and Atopic Dermatitis in Adults: Clinical Study. J. Cosmetol. Trichol..

[86] Cremon C., Stanghellini V., Barbaro M.R., Cogliandro R.F., Bellacosa L., Santos J., Vicario M., Pigrau M., Alonso Cotoner C., Lobo B. (2017). Randomised Clinical Trial: The Analgesic Properties of Dietary Supplementation with Palmitoylethanolamide and Polydatin in Irritable Bowel Syndrome. Aliment. Pharmacol. Ther..

[87] Bonucci M., Raggi R., Vacca R.A. (2020). Polydatin and Its Potential Protective Effect on COVID-19. Clin. Nutr..

