# Supplementary material for: Interference of Polydatin/Resveratrol in the ACE2:Spike Recognition during COVID-19 Infection. A Focus on Their Potential Mechanism of Action through Computational and Biochemical Assays
Source: Biomolecules. 2021 Jul 16;11(7):1048. doi: 10.3390/biom11071048 (PMC8301781; doi:10.3390/biom11071048)
Supplement: Supplementary file 1 [file biomolecules-11-01048-s001.zip › biomolecules-1278284-supplementary.pdf]

SUPPLEMENTARY MATERIALS

of the manuscript

Interference of Polydatin/Resveratrol in the ACE2:Spike recognition during COVID-19 infection. A focus on their potential mechanism of action through computational and biochemical assays

Fulvio Perrella [1], Federico Coppola [1], Alessio Petrone [1], Chiara Platella [1], Daniela Montesarchio [1], Annarita Stringaro [2], Giampietro Ravagnan [3], Maria Pia Fuggetta [3,*], Nadia Rega [1,4,*] and Domenica Musumeci [1,5,*]

[1] *Department of Chemical Sciences, University of Naples Federico II, 80126 Naples, Italy; fulvio.perrella@unina.it (F.P.); federico.coppola@unina.it (F.C.); alessio.petrone@unina.it (A.P.); chiara.platella@unina.it (C.P.); montesar@unina.it (D.M.)*

[2] *National Center for Drug Research and Evaluation, Italian National Institute of Health, 00161 Rome, Italy; annarita.stringaro@iss.it*

[3] *Institute of Translational Pharmacology, Consiglio Nazionale delle Ricerche (CNR), 00133 Rome, Italy; gprav@unive.it*

[4] *Centro di Ricerca Interdipartimentale sui Biomateriali (CRIB), University of Naples Federico II, Piazzale Tecchio, 80125 Napoli, Italy*

[5] *Institute of Biostructures and Bioimages, Consiglio Nazionale delle Ricerche (CNR), 80134 Naples, Italy*

***** *Correspondence: mariapia.fuggetta@ift.cnr.it (M.P.F.); nadia.rega@unina.it (N.R.); domenica.musumeci@unina.it (D.M.); Tel.: +39-06-49934610 (M.P.F.); +39-081-674207 (N.R.); +39-081-674143 (D.M.)*

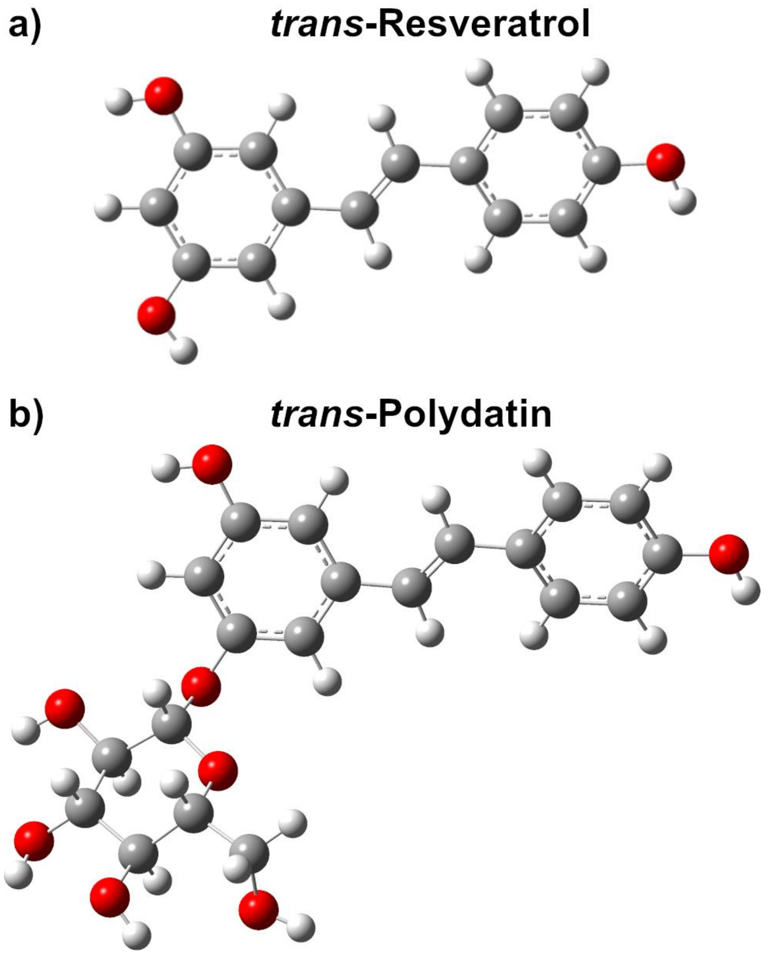

Figure S1. Structures of *trans*-resveratrol (**a**) and *trans*-polydatin (**b**) ligands in ball and stick representation, optimized in gas phase at B3LYP/6-311+G(d,p) theory level. Carbons are reported in gray, hydrogens in white and oxygens in red.

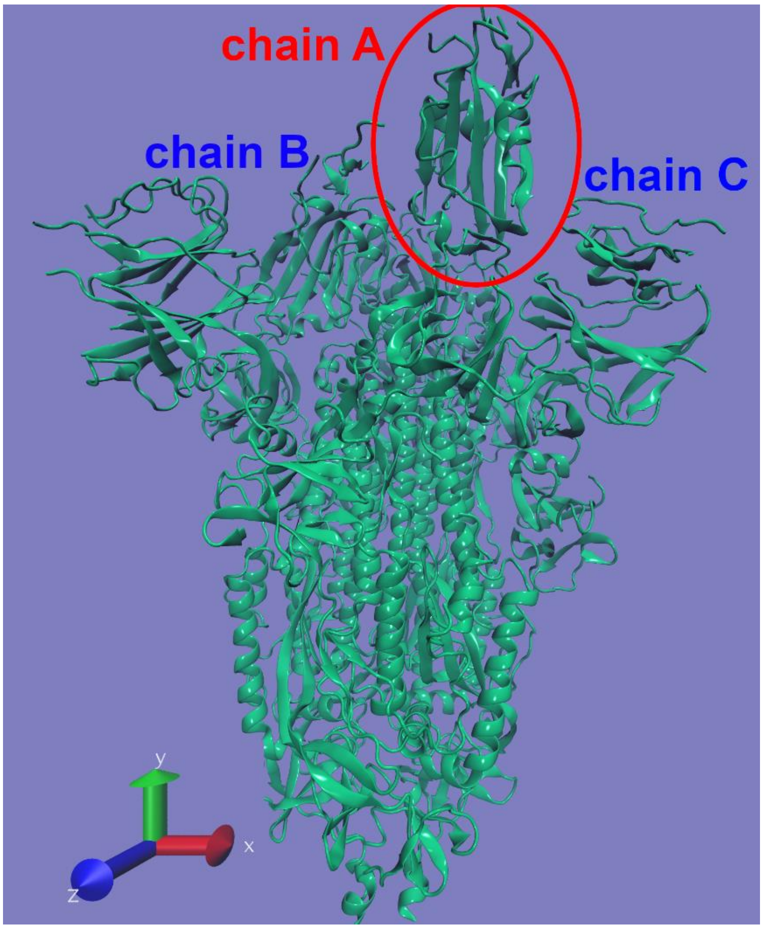

Figure S2. SARS-CoV-2 Spike protein with one RBD in an open conformation (PDB ID: 6VSB). The exposed A-chain RBD, which is the target of RESV and PD docking calculations, is highlighted.

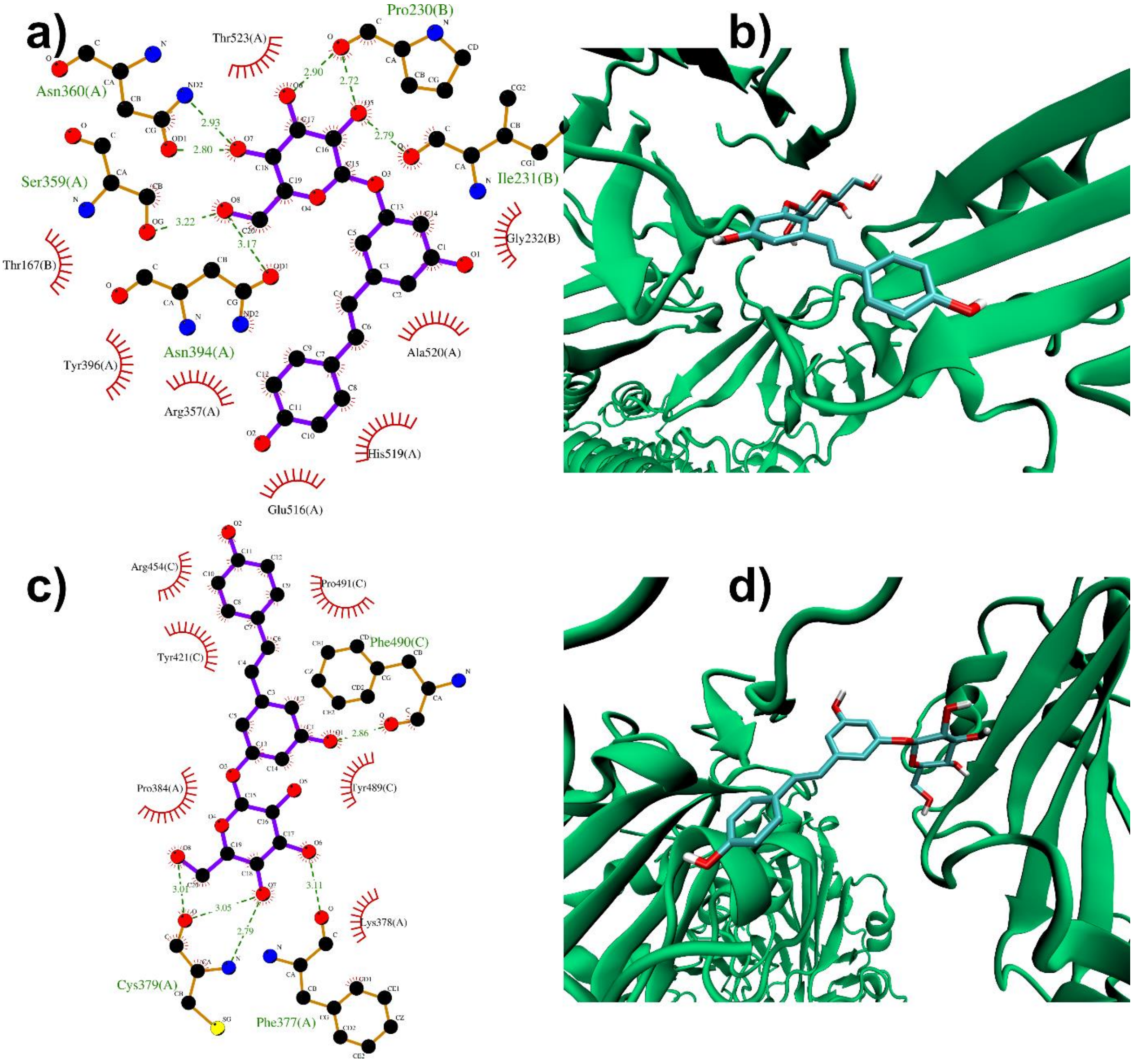

Figure S3. PD top-ranked poses docked to Spike A-chain RBD/B (**a**,**b**) and A-chain RBD/C (**c**, **d**) interfaces. Two-dimensional interaction maps (**a**, **c**): C, N and O atoms are reported in black, blue and red, respectively. Hydrogen bonds are depicted as green dashed lines, while hydrophobic interactions as red cogwheels. Hydrogen atoms are not depicted for ease of illustration. The names of protein residues involved in interactions with the ligand are reported. Three-dimensional representations of PD docked poses (**b**, **d**): Spike backbone is represented as cartoon, while the ligand as sticks.

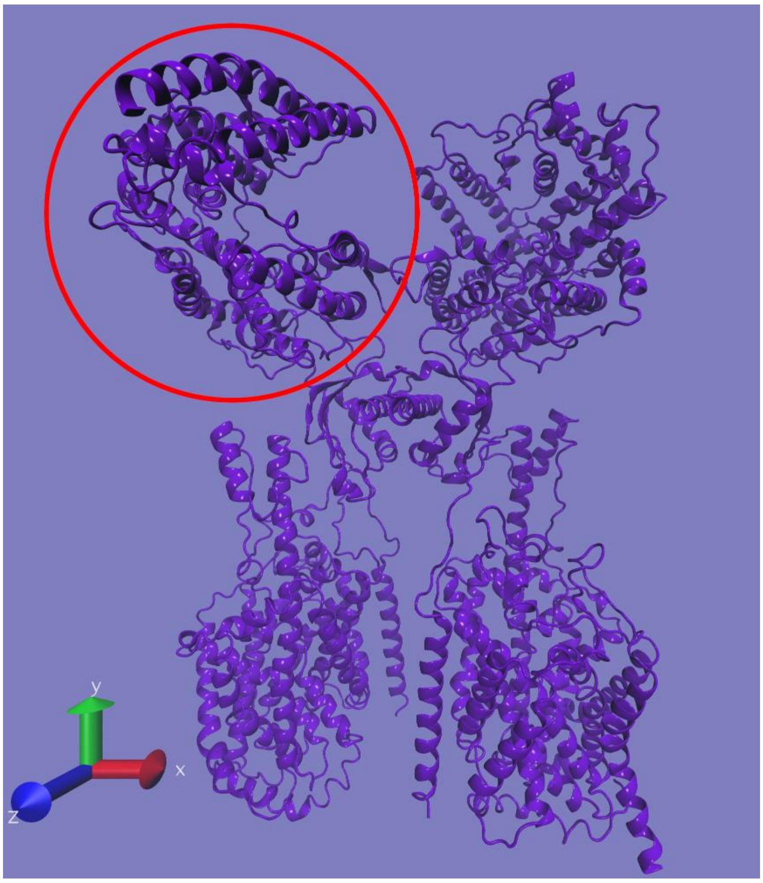

Figure S4. Human ACE2 receptor (PDB ID: 6M18). The protease domain is highlighted.

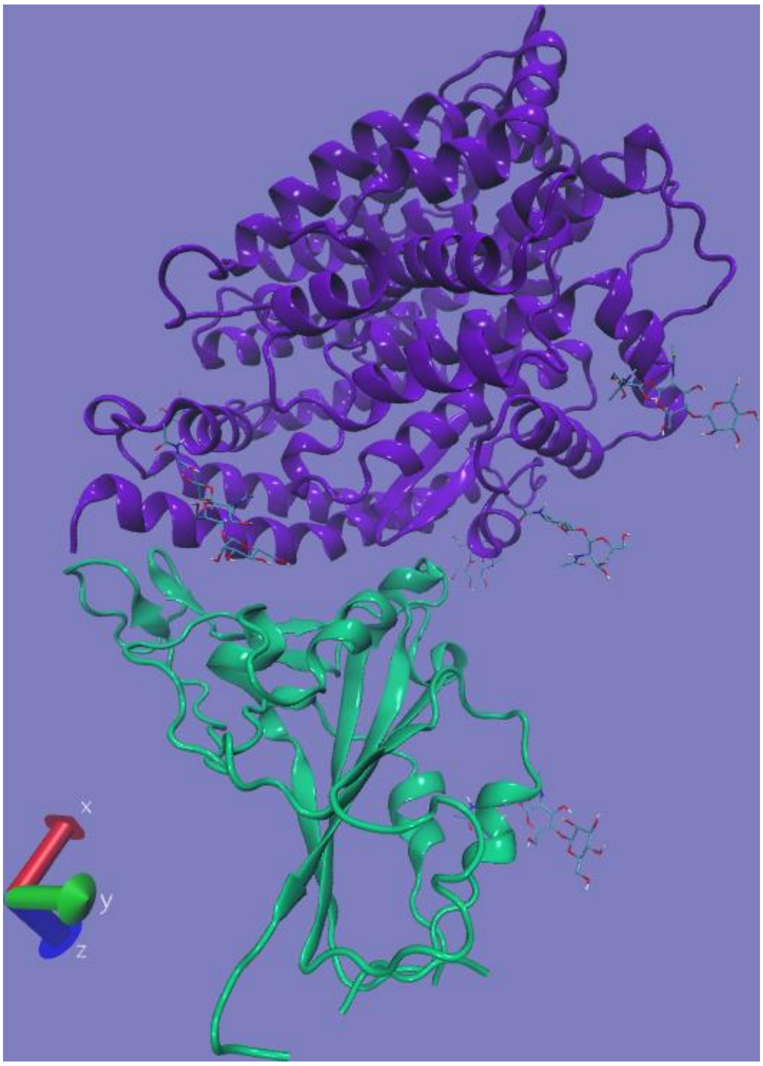

Figure S5. Three-dimensional structure of the SARS-CoV-2 chimeric receptor-binding domain (RBD, green) complexed with its receptor human ACE2 (purple) (PDB ID: 6VW1). Glycosilation sites - BMA:NAG:NAG - are reported as a tube representation.

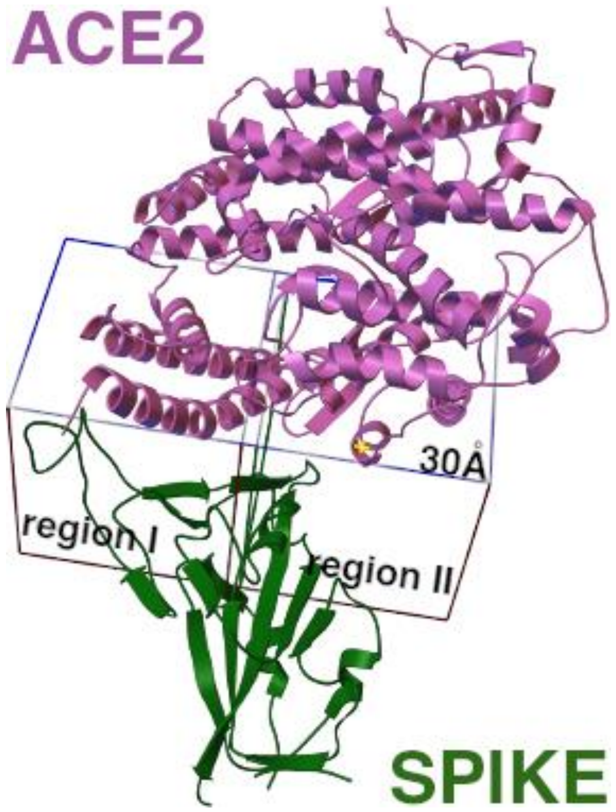

Figure S6. Cubic grids (30 Å per side) used in molecular docking simulations centered on two distinct interfacial regions (regions I and II from left to right, respectively) of the Spike:ACE2 complex.

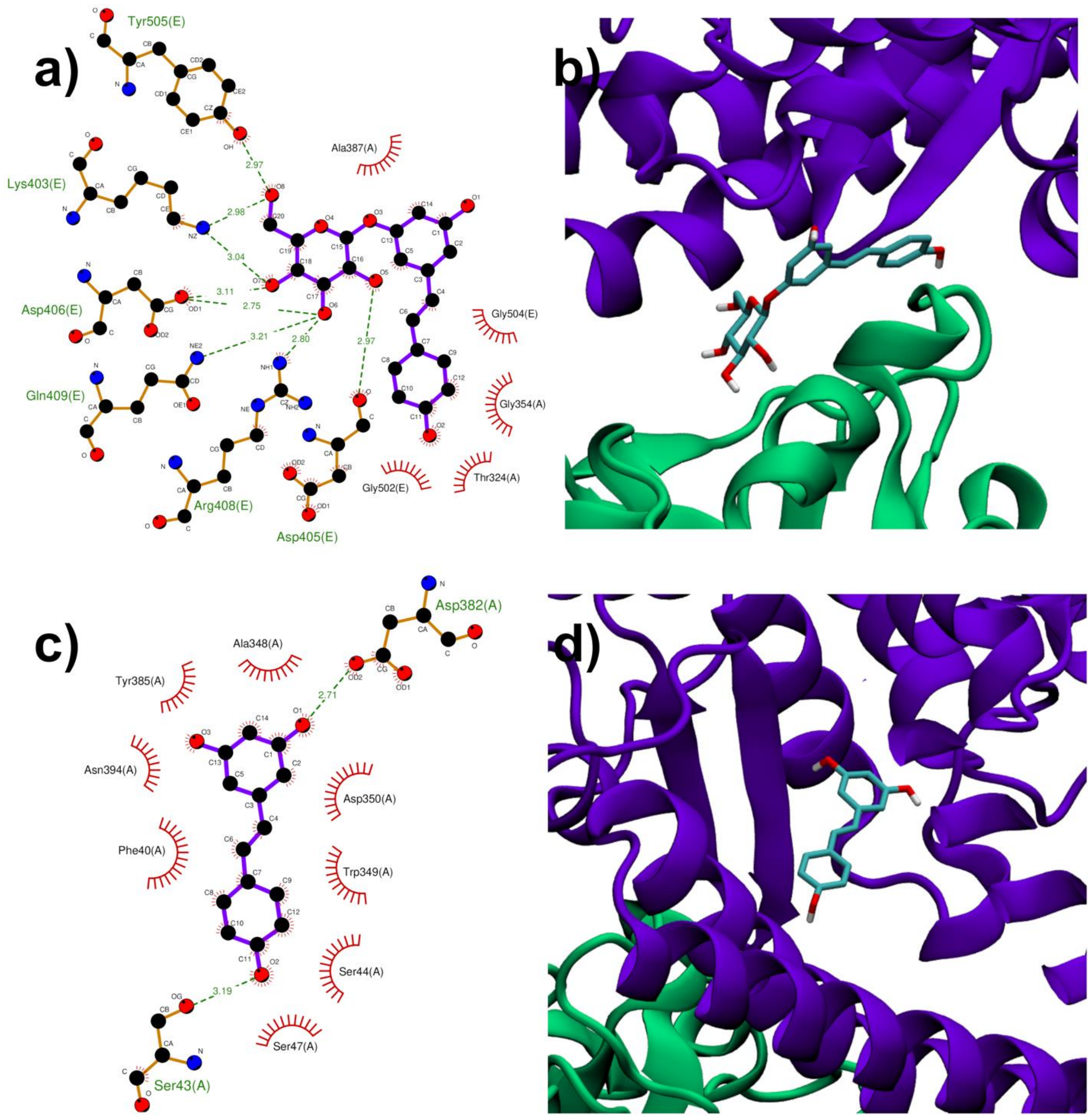

Figure S7. PD (**a**, **b**) and RESV (**c**, **d**) top-ranked poses docked to Spike:ACE2 interface region II.

Table S1. Description of the binding pocket for each PD and RESV docked pose.

	PD	RESV
Spike RBD	Phe342, Ser373, Ser375, Asn437, Arg509, Trp436, Ser438, Asp442, Asn440, Leu441, Ala344, Asn343	Asp405, Arg403, Glu406, Tyr453, Gly496, Tyr495, Gly416, Ile418, Lys417
Spike A/B interface	Thr167(B), Ser359(A), Asn360(A), Thr523(A), Pro230(B), Ile231(B), Gly232(B), Ala520(A), His519(A), Glu516(A), Arg357(A), Asn394(A), Tyr396(A)	
Spike A/C interface	Cys379(A), Pro384(A), Tyr421(C), Arg454(C), Pro491(C), Phe490(C), Tyr489(C), Lys378(A), Phe377(A)	
ACE2	NAG905, Gln102, Gly205, Glu208, Trp566, Asp206, Ala396, Lys562, Leu95, Gln98, Asn194, Tyr196, His195	Tyr196, Gly205, Leu95, Glu208, Glu564, Lys562, Gln98, Gln102
S:ACE2 region I	Val417(E), Asn33(A), His34(A), Glu37(A), Ser494(E), Gly496(E), Tyr495(E), Lys353(A), Tyr505(E), Arg393(A), Pro389(A), Asp30(A)	Pro389(A), Asn33(A), Asp30(A), His34(A), Glu37(A), Asp38(A), Lys353(A), Ser494(E), Gly496(E), Tyr495(E), Lys403(E), Tyr505(E)
S:ACE2 region II	Gly502(E), Asp405(E), Arg408(E), Gln409(E), Asp406(E), Lys403(E), Tyr505(E), Ala387(A), Gly504(E), Gly354(A), Thr324(A)	Ser43(A), Phe40(A), Asn394(A), Tyr385(A), Ala348(A), Asp382(A), Asp350(A), Trp349(A), Ser44(A), Ser47(A)

Table S2. Outputs of docking simulations, showing the first 10 highest-scores poses.

PD, Spike RBD		
Search box center (x,y,z Å)	(206.425, 197.869, 278.290)	
Docked poses	Docking score (kcal/mol)	RMSD (Å)
1	−6.9	0.000
2	−6.7	14.940
3	−6.6	20.895
4	−6.6	14.664
5	−6.6	21.124
6	−6.5	17.289
7	−6.5	16.827
8	−6.4	2.841
9	−6.4	14.571
10	−6.4	16.612
RESV, Spike RBD		
Search box center (x,y,z Å)	(210.804, 200.565, 290.647)	
Docked poses	Docking score (kcal/mol)	RMSD (Å)
1	−6.5	0.000
2	−6.2	0.538
3	−6.2	2.058
4	−6.2	1.584
5	−6.0	23.488
6	−6.0	1.222
7	−5.9	1.900
8	−5.9	23.979
9	−5.8	1.202
10	−5.7	1.177

PD, Spike A/B interface		
Search box center (x,y,z Å)	(225.550, 188.787, 271.544)	
Docked poses	Docking score (kcal/mol)	RMSD (Å)
1	−7.3	0.000
2	−6.9	1.455
3	−6.9	11.695
4	−6.9	8.951
5	−6.9	9.922
6	−6.8	9.999
7	−6.8	9.300
8	−6.8	10.127
9	−6.6	13.741
10	−6.6	10.205
PD, Spike A/C interface		
Search box center (x,y,z Å)	(206.380, 204.679, 278.869)	
Docked poses	Docking score (kcal/mol)	RMSD (Å)
1	−7.3	0.000
2	−7.0	1.087
3	−7.0	4.506
4	−7.0	2.181
5	−6.9	3.376
6	−6.8	2.221
7	−6.8	1.794
8	−6.8	1.875
9	−6.6	1.830
10	−6.5	7.340
PD, ACE2		

Search box center (x,y,z Å)	(148.240, 205.569, 225.218)	
Docked poses	Docking score (kcal/mol)	RMSD (Å)
1	−8.4	0.000
2	−8.1	1.696
3	−8.1	1.863
4	−8.1	1.077
5	−8.0	1.828
6	−7.9	2.559
7	−7.9	5.016
8	−7.9	1.777
9	−7.9	1.701
10	−7.8	4.107
RESV, ACE2		
Search box center (x,y,z Å)	(144.837, 191.919, 232.927)	
Docked poses	Docking score (kcal/mol)	RMSD (Å)
1	−6.9	0.000
2	−6.7	1.936
3	−6.7	0.873
4	−6.5	3.807
5	−6.4	3.780
6	−6.3	1.393
7	−6.3	8.729
8	−6.2	16.435
9	−6.2	16.312
10	−6.2	6.062
PD, Spike:ACE2 region I		

Search box center (x,y,z Å)	(83.196, −22.656, 176.427)	
Docked poses	Docking score (kcal/mol)	RMSD (Å)
1	−8.1	0.000
2	−8.1	2.561
3	−7.5	2.658
4	−7.5	7.569
5	−7.5	2.619
6	−7.4	6.318
7	−7.3	7.627
8	−7.3	1.871
9	−7.2	4.098
10	−7.2	3.666
RESV, Spike:ACE2 region I		
Search box center (x,y,z Å)	(83.196, −22.656, 176.427)	
Docked poses	Docking score (kcal/mol)	RMSD (Å)
1	−7.6	0.000
2	−7.4	1.226
3	−7.3	0.928
4	−7.2	1.388
5	−7.1	3.969
6	−6.8	1.351
7	−6.7	3.758
8	−6.7	4.233
9	−6.5	12.961
10	−6.4	12.781
PD, Spike:ACE2 region II		

Search box center (x,y,z Å)	(83.196, 5.52, 176.427)	
Docked poses	Docking score (kcal/mol)	RMSD (Å)
1	−6.9	0.000
2	−6.8	23.174
3	−6.8	4.773
4	−6.7	3.155
5	−6.7	5.569
6	−6.7	5.999
7	−6.6	9.810
8	−6.6	6.416
9	−6.6	5.812
10	−6.6	5.243
RESV, Spike:ACE2 region II		
Search box center (x,y,z Å)	(83.196, 5.52, 176.427)	
Docked poses	Docking score (kcal/mol)	RMSD (Å)
1	−6.5	0.000
2	−6.4	1.927
3	−6.2	15.757
4	−6.2	2.422
5	−6.2	1.104
6	−6.1	15.896
7	−6.1	2.007
8	−6.0	15.958
9	−5.9	4.279
10	−5.9	15.484

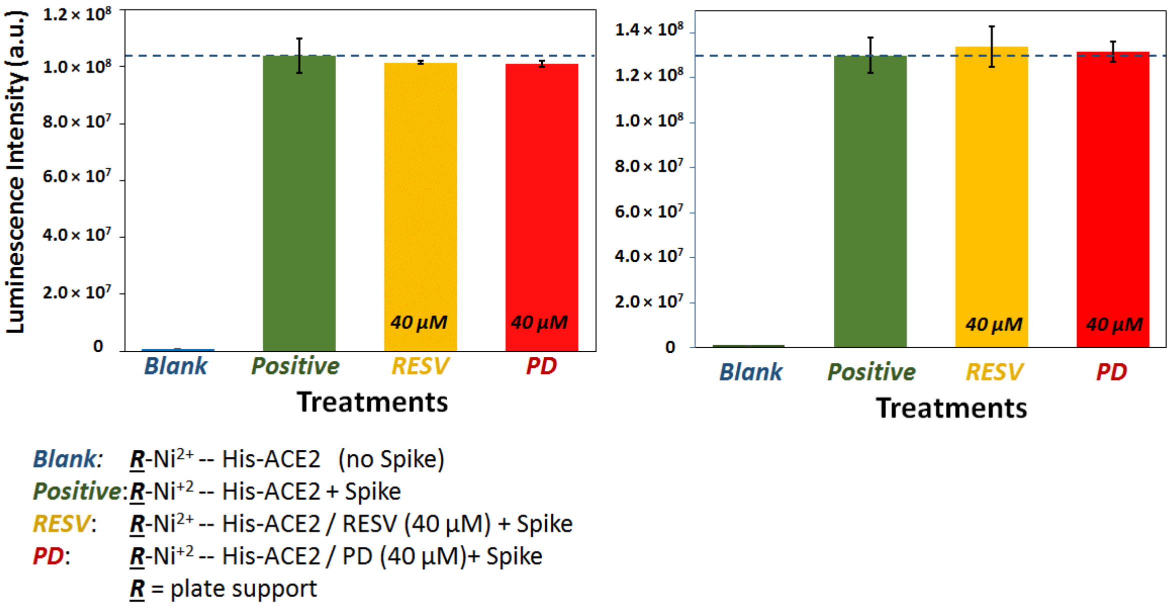

Figure S8. ACE2:Spike Inhibition binding assay. Two independent experiments in which the polyphenols were pre-incubated with ACE2 on the plate before the addition of Spike. Chemiluminescence intensity were measured on the 96-well plate with a luminescence reader.

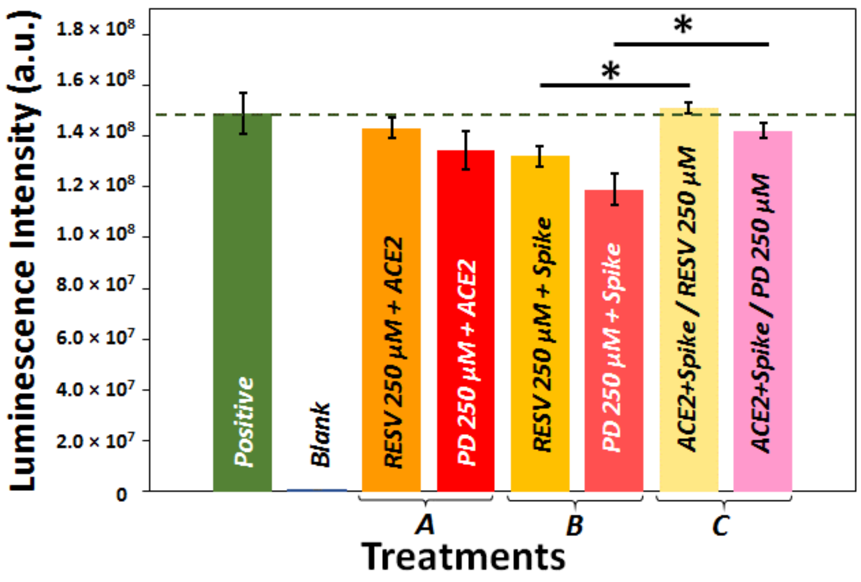

Figure S9. ACE2:Spike inhibition binding assay. In Treatment A, the polyphenols were pre-incubated with ACE2 on the plate, and then Spike was added; in Treatment B, the polyphenols were pre-incubated with Spike in solution, and this mixture was then added to ACE2 on the plate; in Treatment C, ACE2 and Spike were pre-incubated on the plate and then the polyphenols were added. Chemiluminescence intensity was measured on the 96-well plate with a luminescence reader. *p*-Values have been calculated using the Student's t-test ($*p \leq 0.05$).

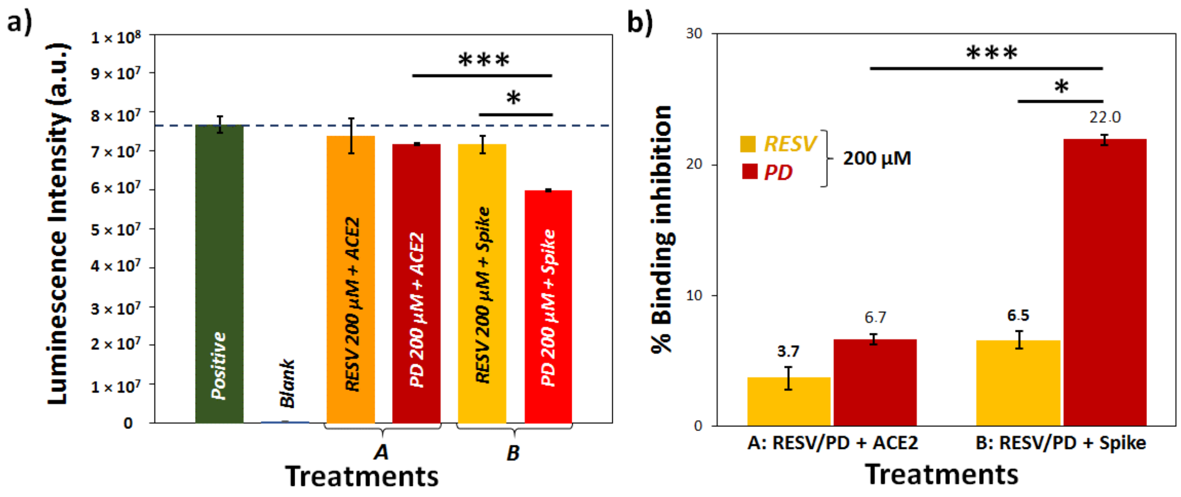

Figure S10. ACE2:Spike Inhibition binding assay. In Treatment A, the polyphenols were pre-incubated with ACE2 on the plate; in Treatment B, the polyphenols were pre-incubated with Spike in solution. **a**) Chemiluminescence intensity measured on the 96-well plate with a luminescence reader; **b**) Complementary percentages, with respect to the positive value, of ACE2:Spike-binding inhibition by RESV and PD. *p*-Values have been calculated using the Student's t-test (*$p \leq 0.05$; ***$p \leq 0.001$).

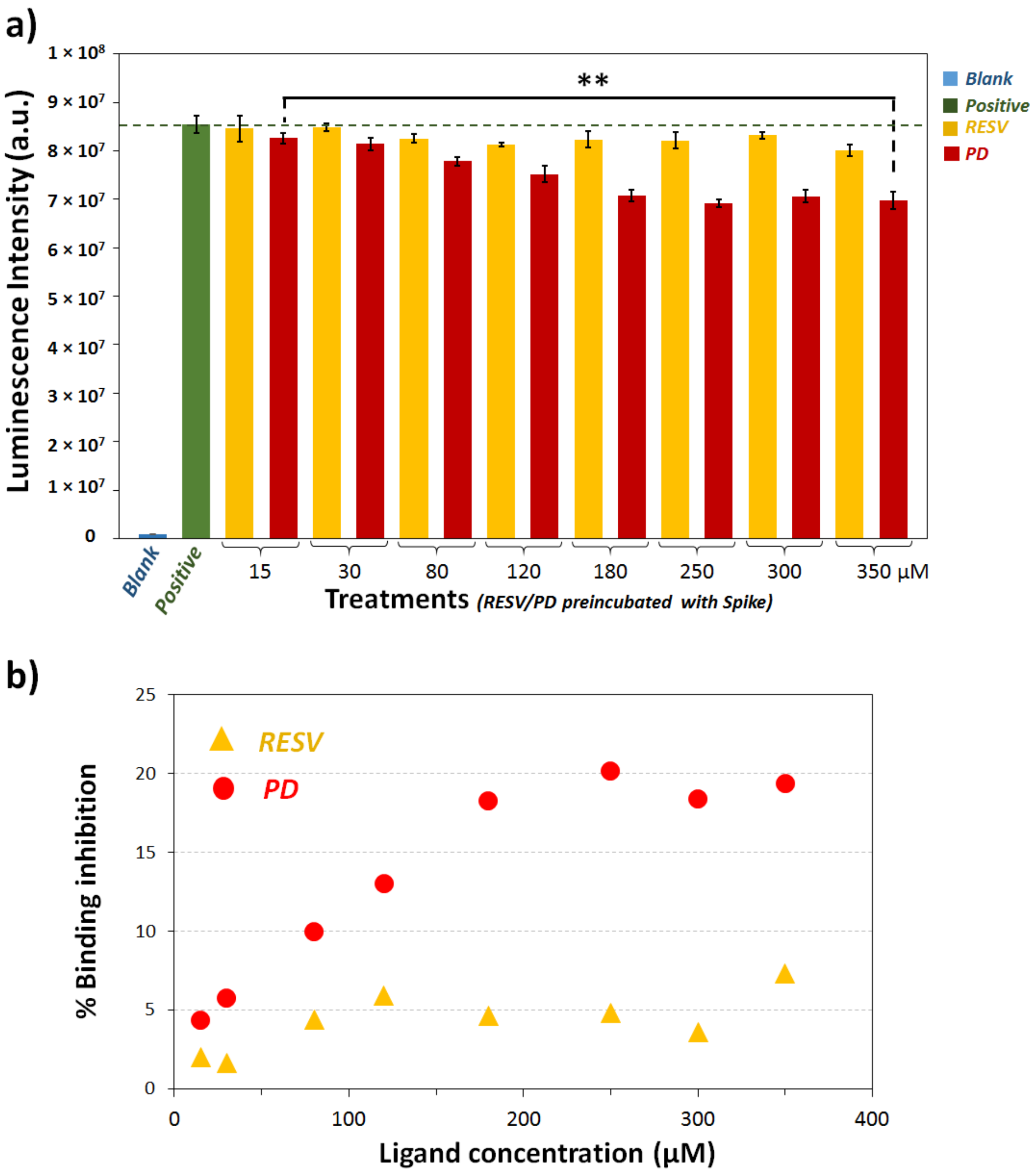

Figure S11. ACE2:Spike inhibition binding assay. In all treatments, the polyphenols were pre-incubated with Spike in solution. **a**) Chemiluminescence intensity was measured on the 96-well plate with a luminescence reader; **b**) Complementary percentages, with respect to the positive-treatment value (in **a**), of ACE2:Spike-binding inhibition by RESV and PD. *p*-Values have been calculated using the Student's t-test (**$p \leq 0.01$).
